# Wnt/β-catenin signalling is dispensable for adult neural stem cell homeostasis and activation

**DOI:** 10.1242/dev.199629

**Published:** 2021-10-19

**Authors:** Sophie H. L. Austin, Rut Gabarró-Solanas, Piero Rigo, Oana Paun, Lachlan Harris, François Guillemot, Noelia Urbán

**Affiliations:** 1 The Francis Crick Institute, London NW1 1AT, UK; 2 Institute of Molecular Biotechnology of the Austrian Academy of Sciences (IMBA), Vienna Biocenter Campus (VBC), Dr. Bohr Gasse 3, 1030 Vienna, Austria; 3 Vienna BioCenter PhD Program, Doctoral School of the University of Vienna and Medical University of Vienna, Vienna A-1030, Austria

**Keywords:** Adult neurogenesis, Adult stem cells, Quiescence, Wnt

## Abstract

Adult mouse hippocampal neural stem cells (NSCs) generate new neurons that integrate into existing hippocampal networks and modulate mood and memory. These NSCs are largely quiescent and are stimulated by niche signals to activate and produce neurons. Wnt/β-catenin signalling acts at different steps along the hippocampal neurogenic lineage, but whether it has a direct role in the regulation of NSCs remains unclear. Here, we used Wnt/β-catenin reporters and transcriptomic data from *in vivo* and *in vitro* models to show that adult NSCs respond to Wnt/β-catenin signalling. Wnt/β-catenin stimulation instructed the neuronal differentiation of proliferating NSCs and promoted the activation or differentiation of quiescent NSCs in a dose-dependent manner. However, deletion of β-catenin in NSCs did not affect either their activation or maintenance of their stem cell characteristics. Together, these results indicate that, although NSCs do respond to Wnt/β-catenin stimulation in a dose-dependent and state-specific manner, Wnt/β-catenin signalling is not cell-autonomously required to maintain NSC homeostasis, which reconciles some of the contradictions in the literature as to the role of Wnt/β-catenin signalling in adult hippocampal NSCs.

## Abstract (precis)

**Summary:** Wnt/β-catenin signalling stimulation promotes the exit from quiescence and differentiation of adult hippocampal neural stem cells but is dispensable for homeostatic neurogenesis in the dentate gyrus of young mice.

## INTRODUCTION

Adult neurogenesis, the continuous process of generating new neurons from neural progenitors throughout life, is predominately restricted to two adult brain regions in mice: the ventricular-subventricular zone (V-SVZ) of the lateral ventricles and the dentate gyrus (DG) of the hippocampus, in which adult-born neurons contribute to olfactory bulb and hippocampal functions, respectively (Bond et al., 2015; Lim and Alvarez-Buylla, 2016; Deng et al., 2010). Adult hippocampal neurogenesis relies on a population of multipotent radial glial-like neural stem cells (NSCs) that have both neurogenic and gliogenic potential (Steiner et al., 2004; Encinas et al., 2011; Bonaguidi et al., 2011; Pilz et al., 2018). The majority of NSCs remain out of the cell cycle in a reversible quiescent state (Doetsch et al., 1999; Andersen et al., 2014; Codega et al., 2014; Urbán et al., 2016). An important regulatory step of adult neurogenesis is the transition of NSCs from quiescence to activation (reviewed by Kempermann et al., 2015). Too little stem cell activation results in an insufficient number of new neurons being generated (Andersen et al., 2014). By contrast, excessive activation generates a transient burst in neurogenesis followed by a sharp decline resulting from the depletion of NSCs, which have limited self-renewal capacity (Paik et al., 2009; Renault et al., 2009). Therefore, tight regulation of the transition between quiescent and active NSC states is crucial to ensure the long-term maintenance of the NSC pool and neurogenesis. Niche-derived signals play an important role in regulating this transition (Fuentealba et al., 2012; Choe et al., 2016; Imayoshi et al., 2010; Lie et al., 2005; Mira et al., 2010; Petrova et al., 2013; Qu et al., 2010). For example, Notch and BMP signalling have an integral role in maintaining NSC quiescence (Lavado and Oliver, 2014; Ables et al., 2010; Bonaguidi et al., 2008; Mira et al., 2010). However, less is known about the niche-derived signals that promote the activation of quiescent NSCs.

Wnt ligands and antagonists are expressed by multiple cell types within the DG niche, including NSCs (Lie et al., 2005; Qu et al., 2013; Jang et al., 2013; Seib et al., 2013). In the absence of Wnt, β-catenin is phosphorylated for degradation by GSK3β (Daugherty and Gottardi, 2007). Upon Wnt ligand binding, β-catenin is stabilised and translocates to the nucleus, where it forms a complex with TCF/LEF transcription factors to activate Wnt/β-catenin target genes, such as *Axin2* (Lustig et al., 2002; Nusse and Clevers, 2017). Wnt/β-catenin signalling is differentially active in cells along the neurogenic lineage and regulates both progenitor proliferation and newborn neuron maturation (Lie et al., 2005; Qu et al., 2013; Wexler et al., 2009; Kuwabara et al., 2009; Seib et al., 2013; Jang et al., 2013; Rosenbloom et al., 2020; Heppt et al., 2020).

Stimulating Wnt/β-catenin signalling in the DG promotes proliferation and neurogenesis (Lie et al., 2005; Seib et al., 2013; Jang et al., 2013). The absence of the Wnt inhibitor Dickkopf (DKK1) results in a specific increase in the generation of neuronally committed intermediate progenitor cells (Seib et al., 2013). However, the absence of another Wnt inhibitor, SFRP3, stimulates proliferation but does not favour a neurogenic lineage choice of NSCs (Jang et al., 2013). Inhibiting Wnt/β-catenin signalling has also yielded contrasting results, because it was found to impair the generation of newborn neurons *in vivo* but induce neuronal differentiation *in vitro* (Qu et al., 2013, 2010; Wexler et al., 2009; Kuwabara et al., 2009; Lie et al., 2005). These discrepancies could result from the use of different experimental approaches to modulate Wnt/β-catenin signalling, such as overexpressing a Wnt ligand versus deleting a Wnt inhibitor, or from the use of *in vitro* versus *in vivo* approaches (Lie et al., 2005; Seib et al., 2013; Jang et al., 2013; Kuwabara et al., 2009; Wexler et al., 2009). The majority of these studies have used systemic or hippocampal-wide modulation of Wnt/β-catenin signalling, which do not discriminate between cell-autonomous and non-cell-autonomous effects; neither do they allow identification of the step(s) in the NSC lineage in which Wnt/β-catenin signalling acts (Lie et al., 2005; Seib et al., 2013; Jang et al., 2013; Qu et al., 2013). In addition, the use of constitutive knockout mice makes it difficult to distinguish between a developmental and an adult neurogenesis phenotype (Seib et al., 2013; Jang et al., 2013; Qu et al., 2013, 2010). Furthermore, earlier studies did not robustly distinguish between NSCs with radial morphology (NSCs) and intermediate progenitor cells (IPCs); therefore the role of Wnt/β-catenin signalling in NSCs, particularly in their transition between active and quiescent states, remains unclear (Kuwabara et al., 2009; Seib et al., 2013).

Here, we used genetic and pharmacological tools to measure and manipulate Wnt/β-catenin signalling specifically in active and quiescent adult NSCs both *in vivo* and *in vitro*. We show that both quiescent and active NSCs respond to Wnt/β-catenin signalling, that the response of NSCs to Wnt/β-catenin stimulation is dose and cell state specific, but that Wnt/β-catenin signalling is not essential for cell-autonomous NSC homeostasis or for the ability of NSCs to proliferate and generate neuronal progeny. Together, these findings reconcile some of the current contradictions relating to the role of Wnt/β-catenin signalling in adult hippocampal NSCs.

## RESULTS

### Quiescent and active NSCs show similar levels of Wnt/β-catenin signalling activity *in vivo*

**Fig. 1.**
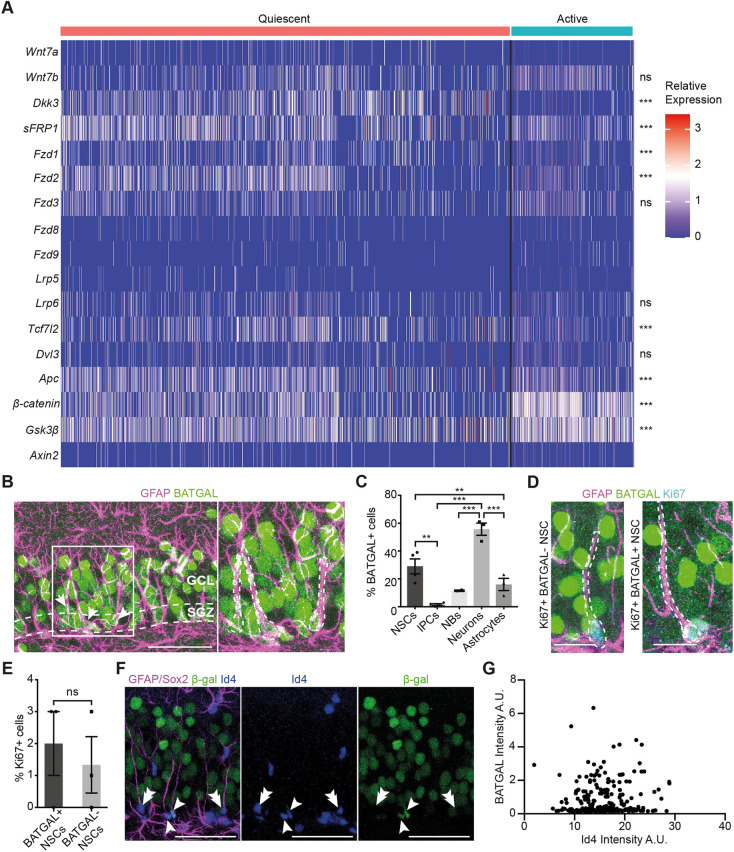
**NSCs *in vivo* respond to Wnt/β-catenin signalling independently of their activation state.** (A) Heatmap of publicly available (GSE159768) single-cell RNA-sequencing data showing the expression of Wnt/β-catenin pathway components from *in vivo* hippocampal quiescent and active NSCs (Harris et al., 2021). (B) BATGAL and GFAP immunolabelling in the DG of a 2-month-old BATGAL Wnt/β-catenin reporter mouse (Maretto et al., 2003). Arrows indicate BATGAL+ NSCs (SGZ cell body and GFAP+ radial process). The white-boxed area is enlarged in the adjacent panel to show three BATGAL+ NSCs. White-dashed lines denote the SGZ. (C) Quantification of the proportion of BATGAL+ cells in the DG of 2-month-old BATGAL mice, showing NSCs (GFAP+ NSCs, 29±5.44%; *n*=4), IPCs (GFAP− SOX2+, 1.5±0.6%; *n*=4), neuroblasts (NBs, DCX+, 11.5±0.5; *n*=2), neurons (NeuN+, 55.67±4.44%, *n*=3) and astrocytes (GFAP+ SOX2−, 16±4.44%, *n*=3). (D) BATGAL immunolabelling in Ki67+ NSCs in 2-month-old BATGAL mice. White-dashed lines denote Ki67+ BATGAL+ NSCs and Ki67+ BATGAL− NSCs. (E) Quantification of the proportion of Ki67+ immunolabelling in BATGAL+ (2±1%) and BATGAL− NSCs (1.33±0.99%) shown in D. *n*=3. (F) BATGAL, GFAP/SOX2 and Id4 immunolabelling in the DG of 2-month-old BATGAL mice. Single arrowheads indicate Id4-positive BATGAL-positive NSCs. Double arrowheads indicate Id4-positive, BATGAL-negative NSCs. (G) Quantification of the data shown in F. *n*=3. Data were analysed as follows: Student's *t*-test (A), ordinary one-way ANOVA with Tukey's multiple comparisons test (C) and unpaired two-tailed Student's *t*-test (E); ns, *P*>0.05, **P<0.01, ***P<0.001. Data are mean±s.e.m. A.U., arbitrary units. Scale bars: 20 µm in D; 50 µm in B,F.

To study the role of Wnt/β-catenin in the transition of hippocampal stem cells from quiescence to activation, we first investigated whether quiescent and active NSCs express components of the Wnt/β-catenin signalling pathway. Although expression of Wnt/β-catenin signalling components in adult hippocampal NSCs has been reported in previously published single-cell sequencing datasets, too few NSCs were identified in most of these datasets to allow a robust comparison between quiescent and active states (Hochgerner et al., 2018). We re-analysed a previously generated single-cell RNA-sequencing dataset containing 2947 NSCs (Fig. 1A) (Harris et al., 2021). We found that both quiescent and active NSCs heterogeneously expressed components of the Wnt/β-catenin signalling pathway, as well as Wnt ligands (*Wnt7a* and *Wnt7b*) and Wnt inhibitors (*Dkk3* and *Sfrp1*), suggesting that NSCs respond to, and directly regulate, Wnt activity levels in the DG (Fig. 1A).

Quiescent NSCs expressed Wnt receptors (*Fzd1* and *Fzd2*) at a higher level compared with active NSCs, which corroborates previous reports showing that quiescent NSCs are enriched for cell surface receptors (Fig. 1A) (Shin et al., 2015; Hochgerner et al., 2018; Artegiani et al., 2017; Cheung and Rando, 2013). The Wnt transducer molecule *Tcf7l2* is more highly expressed in quiescent than in active NSCs, whereas β-catenin (*Ctnnb1*) and GSK3β (*Gsk3b*) are upregulated in active NSCs (Fig. 1A). However, these differences in expression do not translate into a differential expression of Wnt targets between quiescent and active NSCs. *Axin2* was expressed at low levels by very few quiescent (5%) and active (7%) NSCs (Fig. 1A), which might suggest that few NSCs respond to Wnt/β-catenin signalling, but could also reflect the limitation of single cell RNA-sequencing in detecting lowly expressed genes (Haque et al., 2017).

The similar *Axin2* levels between quiescent and active NSCs suggest that cells in these two states respond similarly to Wnt/β-catenin signalling (Fig. 1A). To further characterise the response of NSCs and other DG cells to Wnt/β-catenin signalling *in vivo*, we used β-galactosidase (BATGAL) Wnt/β-catenin reporter mice, which express β-galactosidase (*Glb1*) under the control of 7xTCF/LEF-binding sites (Maretto et al., 2003). We identified NSCs based on their glial fibrillary acidic protein (GFAP) expression, their subgranular zone (SGZ) cell body localisation and their radial process extending through the granule cell layer (GCL) (Fig. 1B). We found that a higher proportion of NSCs (29±5.4%) and neurons (55.7±4.4%) responded to Wnt/β-catenin signalling compared with IPCs (1.5±0.6%), neuroblasts (11.5±0.5%) and astrocytes (16±4.4%) (Fig. 1C), corroborating previous reports (Garbe and Ring, 2012; Heppt et al., 2020). To investigate whether the Wnt/β-catenin response correlates with NSC activation, we quantified proliferation in BATGAL-positive and -negative NSCs (Fig. 1D). The proportion of proliferating NSCs was similar between these two cell states (Fig. 1E). Two different populations can be distinguished within the quiescent NSC pool, with resting NSCs having a higher proliferative potential compared with dormant NSCs (Harris et al., 2021; Urbán et al., 2016). We quantified Id4 immunostaining levels, which are lower in resting compared with dormant NSCs (Harris et al., 2021) alongside BATGAL levels in Id4-positive NSCs (Fig. 1F). Our results showed no correlation between Id4 and BATGAL levels, indicating no difference in Wnt signalling response between resting and dormant NSCs (Fig. 1G). Overall, these data show that, *in vivo*, NSCs respond similarly to Wnt/β-catenin signalling, independently of their activation state.

### NSC maintenance and adult neurogenesis are unaffected by deletion of β-catenin in NSCs *in vivo*

**Fig. 2.**
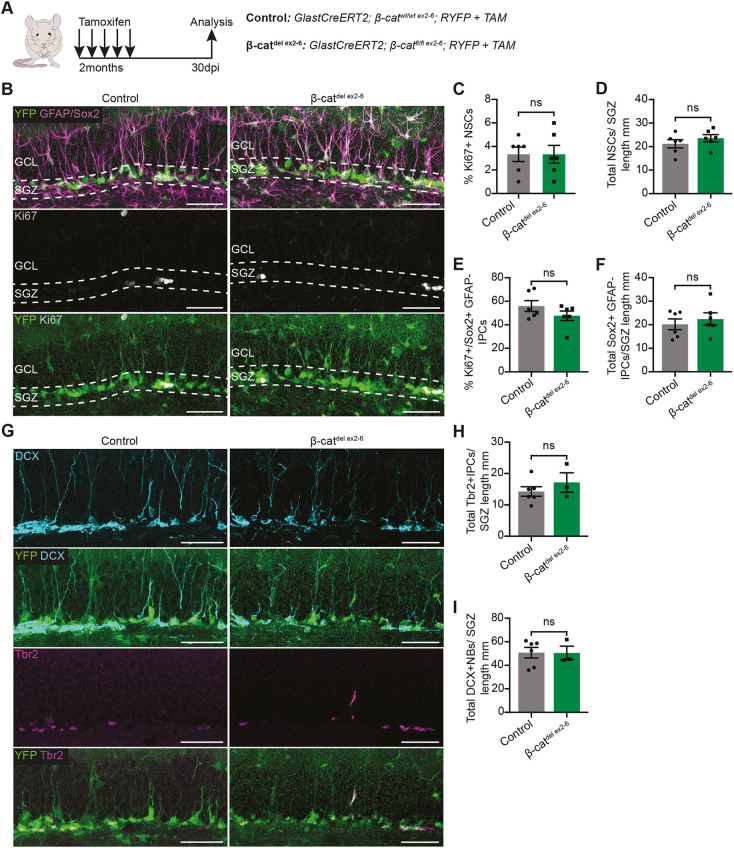
**NSCs and adult hippocampal neurogenesis are unaffected by the NSC-specific deletion of β-catenin and inhibition of Wnt/β-catenin signalling.** (A) Two-month-old control and β-cat^del ex2-6^ mice were administered tamoxifen for 5 consecutive days and sacrificed 30 days after the first tamoxifen injection. (B) YFP, GFAP, SOX2 and Ki67 immunolabelling in the DG of control and β-cat^del ex2-6^ mice 30 days after tamoxifen administration. White-dashed lines denote the SGZ. (C-F) Quantification of the data shown in B. C: proportion of Ki67+ NSCs (control versus β-cat^del ex2-6^: 3.33±0.61% versus 3.333±0.76%). D: total number of NSCs (YFP+ GFAP+ SOX2+ radial cells in the SGZ) normalised to the length of the SGZ (mm) (control versus β-cat^del ex2-6^: 21.2±1.73 versus 23.56±1.52). E: proportion of proliferating (Ki67+) IPCs (SOX2+ YFP+ GFAP− cells in the SGZ; control versus β-cat^del ex2-6^: 56±4.56% versus 47.67±4.03%). F: total number of IPCs normalised to the length of the SGZ (mm) (SOX2+ YFP+ GFAP− cells in the SGZ; control versus β-cat^del ex2-6^: 20.18±2.31 versus 22.43±2.62). *n*=6. (G) YFP, TBR2 and DCX immunolabelling in the DG of control and β-cat^del ex2-6^ mice 30 days after tamoxifen administration. (H,I) Quantification of the data shown in G. H: total number of TBR2+ IPCs normalised to the SGZ length (mm) (TBR2+ YFP+ cells in the SGZ; control versus β-cat^del ex2-6^: 14.29±1.51 versus 17.16±3.09). I: total number of neuroblasts (NBs) normalised to the SGZ length (mm) (DCX+ YFP+ cells; control versus β-cat^del ex2-6^: 50.72±4.49 versus 50.53±5.69). *n*=6 and *n*=3 for control and β-cat^del ex2-6^ mice, respectively. Data analysed using unpaired two-tailed Student's *t*-test; ns, *P*>0.05. Scale bars: 50 µm in B,G. Data are mean±s.e.m.

To investigate the effects of Wnt/β-catenin inhibition in NSCs *in vivo*, we generated *GlastCreERT2; β-cat^fl/fl ex3-6^; RYFP* mice to delete β-catenin conditionally in Glast-expressing NSCs by tamoxifen-inducible, Cre-mediated recombination (Huelsken et al., 2001; Mori et al., 2006; Srinivas et al., 2001). However, the β-catenin allele failed to recombine in NSCs (Fig. S1); therefore, we generated a second β-catenin floxed mouse line: *GlastCreERT2; β-cat^fl/fl ex2-6^; RYFP* mice (hereafter referred to as β-cat^del ex2-6^ mice, Fig. S2A) (Brault et al., 2001). The expression of the recombined *Ctnnb1* transcript was significantly decreased in YFP-positive fluorescence-activated cell (FAC)-sorted cells from the DG of β-cat^del ex2-6^ mice compared with controls (Fig. S2B,C), indicating the successful recombination of the *β-cat^fl/fl ex2-6^* allele. β-catenin protein levels were low in the DG, especially when compared with the SVZ (Fig. S2D). Nevertheless, we were able to detect β-catenin staining in the radial processes of NSCs in control mice, which was eliminated in recombined (YFP-positive) NSCs (Fig. S2E,F), confirming the deletion of β-catenin protein. However, *Axin2* transcript levels were very low and unchanged between YFP-positive FAC-sorted cells from the DG of β-cat^del ex2-6^ mice compared with controls (Fig. S2G). Given that beta-galactosidase accumulates in the cell, it can be a more-sensitive readout of Wnt activity. Therefore, we crossed β-cat^del ex2-6^ mice with BATGAL mice and found that the proportion of BATGAL-positive NSCs was reduced in β-cat^del ex2-6^ mice compared with controls, confirming that the loss of β-catenin impairs Wnt/β-catenin activity in NSCs (Fig. S2H-J). To examine the effect of β-catenin deletion on NSCs, we administered tamoxifen to 2-month-old β-cat^del ex2-6^ and control mice and performed immunofluorescence analysis 30 days later, focusing on recombined YFP+ cells (Fig. 2A,B). The proportion of Ki67-positive NSCs was not significantly different between β-cat^del ex2-6^ and control mice, indicating that β-catenin deletion did not perturb NSC proliferation (Fig. 2C). We also quantified the total number of NSCs 30 days (Fig. 2D) and 90 days (Fig. S3A,B) after tamoxifen administration and found no significant difference between genotypes, indicating that NSC maintenance was unaffected by β-catenin deletion. Overall, these data suggest that the proliferation and maintenance of NSCs are unaffected by loss of Wnt/β-catenin signalling.

We then investigated how the loss of β-catenin affects later steps in adult hippocampal neurogenesis. We quantified the percentage of proliferating IPCs (Fig. 2E), the total number of IPCs (Fig. 2F-H) and the total number of neuroblasts (Fig. 2G,I), and did not find any significant difference between β-cat^del ex2-6^ and control mice 30 days after β-catenin deletion. We also quantified the total number of neuroblasts and newly generated neurons 90 days after tamoxifen administration and again observed no difference between genotypes (Fig. S3C-G). Overall, these data show that loss of Wnt/β-catenin signalling in NSCs *in vivo* does not impair the behaviour or maintenance of NSCs; neither does it impair the generation and survival of new neurons in the adult hippocampus.

### Stabilising β-catenin in NSCs causes their displacement and loss from the DG niche

After having assessed the effects of disrupting Wnt/β-catenin signalling in NSCs *in vivo*, we investigated how stimulating Wnt/β-catenin signalling would affect NSCs. To do so, we generated *GlastCreERT2; Catnb^lox(ex3)/wt^ RYFP* mice [hereafter referred to as Catnb^del(ex3)^ mice] to stabilise β-catenin conditionally in Glast-expressing NSCs (Harada et al., 1999). *Catnb^lox(ex3)^* is a conditional constitutively active allele of β-catenin (*Catnb*) in which exon 3, which encodes the GSK3β phosphorylation sites that mark β-catenin for degradation, is flanked by LoxP sites (Harada et al., 1999). Cre-mediated recombination of the *Catnb^lox(ex3)^* allele resulted in β-catenin stabilisation and ligand-independent activation of downstream Wnt/β-catenin signalling in targeted cells. Upon recombination, we observed an increase in both β-catenin levels and in the intensity of the BATGAL reporter in *Catnb^lox(ex3)^*/BATGAL mice compared with BATGAL controls (Fig. S4A-E).

We injected 2-month-old Catnb^del(ex3)^ and control mice with tamoxifen for 5 consecutive days and analysed their NSCs 10 and 30 days later (Fig. S4A). We first assessed the effect of stabilising β-catenin in NSCs by quantifying the total number of NSCs in Catnb^del(ex3)^ and control mice (Fig. S4F-K). We found a significant decrease in the number of NSCs in Catnb^del(ex3)^ mice compared with controls at 30 days (Fig. S4G) but not at 10 days after tamoxifen administration (Fig. S4J), suggesting that stabilising β-catenin causes NSC loss between 10 and 30 days later. This could be the result of increased NSC proliferation and subsequent depletion. We observed an increase in the proportion of Ki67-positive NSCs 30 days but not 10 days after tamoxifen administration (Fig. S4H,K). Therefore, the loss of NSCs at 30 days could be because of increased proliferation between the 10 and 30 day time points. However, we noticed that the cellular organisation of the DG was already disrupted in Catnb^del(ex3)^ mice 10 days after β-catenin stabilisation. Many YFP-positive recombined cells were displaced throughout the GCL and molecular layer (ML) (Fig. S4I). Some of these cells retained NSC characteristics (GFAP- and SOX2-positive cells with radial morphology) but their cell bodies were not correctly located in the SGZ (Fig. S4L). Such cells were also present in control mice, but the proportion of displaced NSCs was increased three-fold in Catnb^del(ex3)^ compared with control mice (Fig. S4M,N). This suggests that stabilising β-catenin in NSCs promotes their displacement from their correct SGZ location, which might cause their subsequent loss from the DG. Given that β-catenin regulates cell adhesion at adherens junctions (Bienz, 2005), this displacement phenotype could result from disrupted cell adhesion, which precludes using this mouse model to investigate the effects of stimulating Wnt/β-catenin signalling in NSCs *in vivo*. As an alternative approach, we used an established *in vitro* model of hippocampal NSCs that allows manipulation of their quiescent and active states in a niche-independent setting (Blomfield et al., 2019).

### Quiescent and active NSCs *in vitro* show similar levels of Wnt/β-catenin signalling activity

**Fig. 3.**
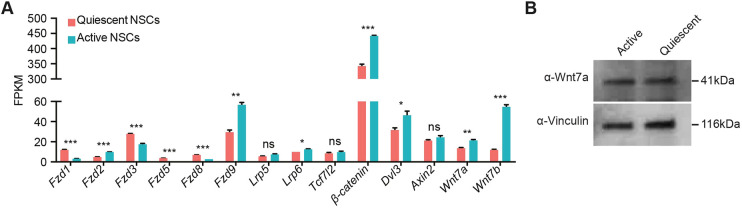
**NSCs in quiescent and active culture conditions express components of the Wnt/β-catenin signalling pathway.** (A) Gene expression data from publicly available (GSE116997) bulk RNA-sequencing analysis comparing the expression levels of Wnt receptors, transducer molecules, the Wnt/β-catenin target gene *Axin2* and Wnt ligands in quiescent and active NSC cultures. *n*=3. (B) Wnt7a western blot in active and quiescent NSC lysates shows that active and quiescent NSCs produce Wnt protein. Data analysed using unpaired two-tailed Student's *t*-test; ns, *P*>0.05, *P<0.05, **P<0.01, ***P<0.001. Data are mean±s.e.m.

Dissociated hippocampal NSCs in adherent cultures can be maintained in a proliferative state by the presence of Fgf2 and can be induced into a reversible state of quiescence through the addition of Bmp4 (Blomfield et al., 2019; Martynoga et al., 2013; Mira et al., 2010). Using a published bulk RNA-sequencing dataset (Blomfield et al., 2019), we found that NSCs in this *in vitro* model system largely recapitulated the expression of Wnt pathway components observed in quiescent and active NSCs *in vivo* (Fig. 3A), including the upregulation of Wnt receptors in quiescent NSCs compared with active NSCs, corroborating published reports (Lie et al., 2005; Wexler et al., 2009). Moreover, quiescent and active NSCs expressed Wnt ligands at both the RNA and protein levels (Fig. 3A,B), suggesting that they can self-regulate their behaviour by autocrine/paracrine Wnt/β-catenin signalling, as previously proposed (Qu et al., 2013, 2010; Wexler et al., 2009). As with NSCs *in vivo*, we found that quiescent and active NSCs *in vitro* expressed *Axin2* at similarly low levels, suggesting that their response to Wnt/β-catenin signalling is independent of their activation state (Fig. 3A).

### Loss of Wnt/β-catenin signalling has no effect on the maintenance of NSC activation states or stem cell potency *in vitro*

We next took advantage of this *in vitro* system to investigate the role of Wnt/β-catenin signalling specifically in active or quiescent NSCs as well as in their transition between states and to determine whether Wnt/β-catenin is dispensable for NSCs *in vitro*, as we demonstrated *in vivo*. We derived an NSC cell line from *β-cat^fl/fl ex3-6^*; RYFP mice (Huelsken et al., 2001; Srinivas et al., 2001), hereafter referred to as β-cat^del ex3-6^ NSCs. When β-cat^del ex3-6^ NSCs were transduced with a Cre-expressing adenovirus, Cre-recombined cells were identified by their expression of YFP. Expression of the recombined portion of the *β-cat* transcript had decreased by 24 h after Cre-transduction and β-catenin protein had become undetectable by 48 h, indicating successful recombination of the *β-cat^fl/fl ex3-6^* allele (Fig. S5A,B). We next examined *Axin2* levels in β-cat^del ex3-6^ and control NSCs treated with CHIR99021, which stimulates Wnt/β-catenin signalling by inhibiting GSK3β (Ring et al., 2003). CHIR99021 treatment upregulated *Axin2* expression levels in control NSCs but not in β-cat^del ex3-6^ NSCs, confirming that deletion of β-catenin impairs the response of NSCs to Wnt/β-catenin signalling (Fig. S5C). Of note, the basal levels of *Axin2* did not change upon β-catenin deletion, indicating that Wnt activity is very low in proliferating NSCs *in vitro* as it is *in vivo*.

**Fig. 4.**
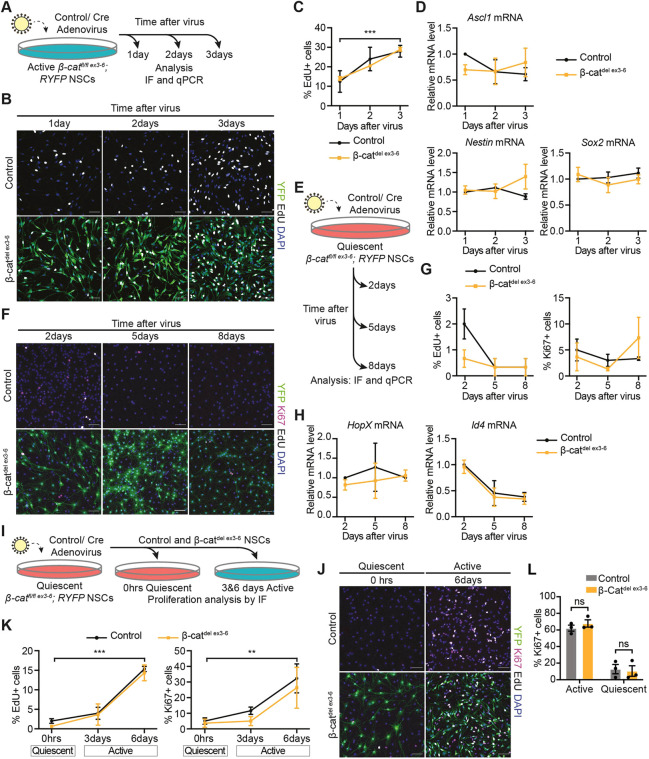
**NSC behaviours are unaffected by loss of β-catenin and impaired Wnt/β-catenin signalling *in vitro*.** (A) Active β-cat^fl/fl ex3-6^ NSCs were transduced with control or Cre-adenovirus and samples were collected 1, 2 and 3 days later. (B) YFP, β-catenin, EdU and DAPI immunolabelling 1, 2 and 3 days after control- and Cre-adenovirus transduction in control and β-cat^del ex3-6^ active NSCs. YFP immunolabelling identifies recombined β-cat^del ex3-6^ NSCs. (C) Quantification of the proportion of EdU+ cells shown in B. In β-cat^del ex3-6^ active NSCs, the proportion of EdU+ cells was calculated as a fraction of the YFP+ recombined cells (control versus β-cat^del ex3-6^: 1 day=12.5±5.5% versus 14±1%; 2 days=24±6% versus 20.5±0.5%; 3 days=28±3% versus 29±1%). The increase in the proportion of proliferating cells was statistically significant across time. *n*=2. (D) Expression of the stem cell-associated genes *Ascl1*, nestin (*Nes*) and *Sox2* in β-cat^del ex3-6^ active NSCs compared with controls 1, 2 and 3 days after virus transduction. *n*=3. (E) Quiescent β-cat^fl/fl ex3-6^ NSCs were transduced with control or Cre-adenovirus following 72 h quiescence induction and samples were collected 2, 5 and 6 days later. (F) YFP, EdU, Ki67 and DAPI immunolabelling 2, 5 and 8 days after control- and Cre-adenovirus transduction in control and β-cat^del ex3-6^ quiescent NSCs. (G) Quantification of the proportion of EdU+ cells (control versus β-cat^del ex3-6^: 2 days=2±0.58% versus 0.67±0.33%; 5 days=0.33±0.33% versus 0.33±0.33%; 8 days=0.33±0.33% versus 0.33±0.33%) and Ki67+ cells (control versus β-cat^del ex3-6^: 2 days=5±2.08% versus 3.67±2.67%; 5 days=3±1.16% versus 1.33±0.33%; 8 days=3.33±0.33% versus 7.33±3.93%) in D. *n*=3. (H) Expression of the quiescence-associated genes *HopX* and *Id4* in control and β-cat^del ex3-6^ quiescent NSCs. *n*=3. (I) Two days after virus transduction of quiescent control and β-cat^fl/fl ex3-6^ NSCs, samples were collected before (0 h) and 3 and 6 days after retuning cells to proliferative culture conditions for immunofluorescence (IF) analysis of proliferation markers. (J) YFP, EdU, Ki67 and DAPI immunolabelling of quiescent (0 h) control and β-cat^del ex3-6^ NSCs and 3 and 6 days after returning the cells to proliferative culture conditions. (K) Quantification of the data shown in J of the proportion of EdU+ cells (control versus β-cat^del ex3-6^: 0 h=2±0.58% versus 0.67±0.33%; 3 days=4±1.53% versus 3.67±2.73%; 6 days=15.33±0.67% versus 14.33±2.03%) and Ki67+ cells (control versus β-cat^del ex3-6^: 0 h=5±2.08% versus 3.67±2.67%; 3 days=11.67±2.33% versus 5±2.89%; 6 days=32.33±9.28% versus 26.33±13.09%). The increase in the proportion of proliferating cells was statistically significant across time. *n*=3. (L) Quantification of the proportion of Ki67+ cells in active control and β-cat^del ex3-6^ NSCs before and after BMP4-induced quiescence (active: control NSCs, 62.16±3.6%, β-cat^del ex3-6^ NSCs, 67.78±4.39%; quiescent: control NSCs, 12.77±5.59%, β-cat^del ex3-6^ NSCs, 10.5±6.36%). *n*=3. Data analysed with two-way ANOVA with Sidak's multiple comparisons test (C,D,G,H,K) and unpaired two-tailed Student's *t*-test (L); ns, *P*>0.05. **P<0.01. ***P<0.001. Data are mean±s.e.m. Scale bars: 50 µm in B,F,J.

We then asked whether the loss of β-catenin, and the resulting inhibition of Wnt/β-catenin signalling, affects the stem cell identity and proliferation of NSCs. Control and β-cat^del ex3-6^ active NSCs were pulsed with EdU for 1 h to label cells in S-phase of the cell cycle at different time points after Cre transduction (Fig. 4A). The proportion of EdU-positive cells was unchanged between control and β-cat^del ex3-6^ active NSCs at all time points analysed (Fig. 4B,C). The stem cell-associated genes, *Ascl1*, nestin (*Nes*) and *Sox2* were similarly expressed between β-cat^del ex3-6^ and control active NSCs at all time points (Fig. 4D). These results suggest that loss of β-catenin does not affect the proliferation or maintenance of NSC identity under these conditions.

We next assessed how the loss of Wnt/β-catenin signalling affects the ability of NSCs to maintain quiescence. To do this, we cultured NSCs under quiescent conditions and then added Cre or Null virus to generate β-cat^del ex3-6^ and control cultures, respectively (Fig. 4E). We found that both cultures maintained a similarly low rate of proliferation over time (Fig. 4F,G). Moreover, the quiescence-associated genes *Hopx* and *Id4* were similarly expressed in control and β-cat^del ex3-6^ quiescent NSCs (Fig. 4H). Overall, these data suggest that BMP-induced NSC quiescence is unaffected by inhibition of Wnt/β-catenin signalling.

So far, we have shown that Wnt/β-catenin signalling is not required to maintain established active or quiescent states of NSCs. However, the role of Wnt/β-catenin signalling in enabling the transition between states (i.e. the activation of quiescent NSCs) remains unaddressed. To investigate this, we returned β-cat^del ex3-6^ and control quiescent NSCs to proliferative culture conditions to promote reactivation (Fig. 4I). We measured proliferation immediately before (time zero) and at 3 and 6 days after returning the cells to proliferative culture conditions (Fig. 4I,J). Proliferation increased similarly in both β-cat^del ex3-6^ and control NSCs, as shown by comparable increases in the proportions of EdU-positive and Ki67-positive cells (Fig. 4K). We also found no difference in the ability of β-cat^del ex3-6^ and control active NSCs to enter BMP-induced quiescence, as shown by the similar proportions of Ki67-positive NSCs between control and β-cat^del ex3-6^ NSCs before and after quiescence induction (Fig. 4L). This suggests that loss of intact Wnt/β-catenin signalling does not affect the transitions of NSCs between quiescent and active states.

Given that NSC proliferation and activity were not affected by loss of Wnt/β-catenin signalling, we next tested whether neuronal or astrocytic differentiation was affected by the chronic loss of β-catenin. We changed the media conditions of β-cat^del ex3-6^ and control active NSCs at 6-, 12- and 18-days after Cre transduction to promote neuronal differentiation (B27 for 5 days) or astrocytic differentiation (FBS for 5 days) (Fig. S6A). We then immunolabelled cells for the neuronal marker MAP2 and astrocyte marker GFAP to identify and quantify the proportions of neurons and astrocytes, respectively (Fig. S6B-E). We found that β-cat^del ex3-6^ NSCs generated a similar proportion of MAP2-positive neurons to control NSCs (Fig. S6C). The proportions of GFAP-positive astrocytes generated were also similar between β-cat^del ex3-6^ and control NSCs (Fig. S6E). This suggests that the neurogenic and gliogenic potential of NSCs is unaffected by the loss of β-catenin-dependent Wnt signalling under these differentiating conditions *in vitro*.

### Wnt/β-catenin stimulation promotes the neuronal differentiation of active NSCs

**Fig. 5.**
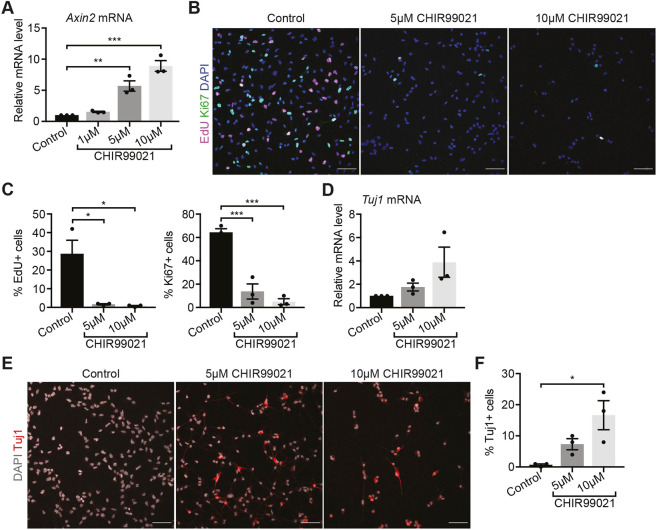
**Stimulating Wnt/β-catenin signalling promotes neuronal differentiation of active NSCs.** (A) Upregulation of *Axin2* expression in active NSCs treated with 5 µM and 10 µM CHIR99021 for 48 h confirms stimulation of Wnt/β-catenin signalling. *n*=3. (B) Immunolabelling of proliferation markers EdU and Ki67 and of DAPI in active NSCs treated with CHIR99021. (C) Quantification of the data shown in B: proportion of EdU+ cells (control, 28.67±7.27%; 5 µM CHIR99021, 1.67±0.33%; 10 µM CHIR99021, 0.67±0.33%) and Ki67+ cells (control, 64.33±3.18%; 5 µM CHIR99021, 13.67±6.49%; 10 µM CHIR99021, 5±2.52%). *n*=3. (D) *Tuj1* expression in control and 10 µM CHIR99021-treated active NSCs (*P*=0.06; *n*=3). (E) Immunolabelling of TUJ1 and DAPI in active NSCs treated with CHIR99021 compared with controls. (F) Quantification of the data shown in E: TUJ1+ cells (control, 0.67±0.33%; 5 µM CHIR99021, 7.33±1.76%; 10 µM CHIR99021, 16.67±4.67%). *n*=3. Data analysed with repeated measures one-way ANOVA with Dunnett's multiple comparison test; ns, *P*>0.05, *P<0.05, **P<0.01, ***P<0.001). Data are mean±s.e.m. Scale bars: 50 µm in B,E.

We next investigated the consequences of stimulating Wnt/β-catenin signalling in NSC cultures. For this, we treated active NSCs with the GSK3β inhibitor CHIR99021 for 48 h. CHIR99021 treatment resulted in a dose-dependent increase in *Axin2* expression levels, which were significantly higher compared with control cells in response to treatment with 5 µM and 10 µM CHIR99021 (Fig. 5A).

We then investigated whether 5 µM and 10 µM CHIR99021 treatments led to changes in the proliferation of active NSCs, and found that the proportions of EdU-positive and of Ki67-positive NSCs decreased in active NSCs treated with CHIR99021 compared with controls (Fig. 5B,C). Given that reduced proliferation could be the result of increased differentiation of CHIR99021-treated NSCs, we next examined the expression of the neuronal marker β-III-tubulin [*Tuj1* (*Tubb3*)]. We found that the expression of *Tuj1* as well as the proportion of TUJ1-positive immunolabelled cells were increased in active NSCs upon 10 µM CHIR99021 treatment (Fig. 5D-F). Overall, these results show that stimulating Wnt/β-catenin signalling in active NSCs reduces their proliferative capacity and promotes neuronal differentiation.

### Wnt/β-catenin stimulation promotes the activation and differentiation of quiescent NSCs in a dose-dependent manner

**Fig. 6.**
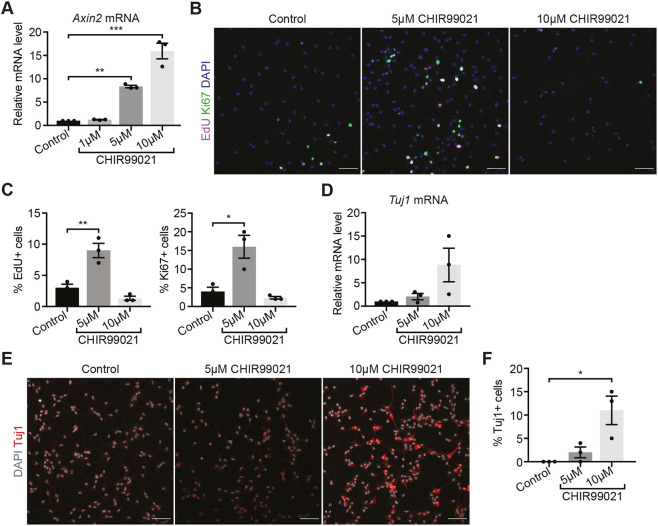
**Stimulating Wnt/β-catenin signalling has dose-dependent effects in quiescent NSCs.** (A) *Axin2* expression in quiescent NSCs treated with 5 µM and 10 µM CHIR99021 for 48 h confirms the dose-dependent stimulation of Wnt/β-catenin signalling. *n*=3. (B) Immunolabelling of the proliferation markers EdU and Ki67, and DAPI in quiescent NSCs treated with CHIR99021 compared with controls. (C) Quantification of the data shown in B: EdU+ cells (control, 3±0.58%; 5 µM CHIR99021, 9±1.16%; 10 µM CHIR99021, 1.33±0.33%) and Ki67+ cells (control, 4±1.16%; 5 µM CHIR99021, 16±3.06%; 10 µM CHIR99021, 2.33±0.33%). *n*=3. (D) *Tuj1* expression in control and 10 µM CHIR99021-treated quiescent NSCs (*P*=0.07; *n*=3). (E) Immunolabelling of TUJ1 and DAPI in quiescent NSCs treated with CHIR99021 compared with controls. (F) Quantification of the data shown in E: proportion of TUJ1+ cells (control, 0±0%; 5 µM CHIR99021, 2±1.16%; 10 µM CHIR99021, 11±3.06%). *n*=3. Data analysed with repeated measures one-way ANOVA with Dunnett's multiple comparison test: ns, *P*>0.05, *P<0.05, **P<0.01, ***P<0.001. Data are mean±s.e.m. Scale bars: 50 µm in B,E.

To investigate the effect of stimulating Wnt/β-catenin signalling in quiescent NSCs, we similarly treated quiescent NSCs with 1 µM CHIR99021, 5 µM CHIR99021 and 10 µM CHIR99021 for 48 h. Using *Axin2* expression levels as a readout of Wnt/β-catenin signalling levels, we found that, similarly to active NSCs, *Axin2* expression was upregulated in a dose-dependent manner by CHIR99021 in quiescent NSCs (Fig. 6A).

We then examined the effects of stimulating Wnt/β-catenin signalling on the proliferation and differentiation of quiescent NSCs. Treating quiescent NSCs with the highest concentration of CHIR99021 (10 µM) did not reduce the already very low levels of proliferation of quiescent NSCs (Fig. 6B,C). However, 10 µM CHIR99021 treatment induced their neuronal differentiation, as seen by the increase in *Tuj1* expression and percentage of TUJ1-positive cells (Fig. 6D-F), which resembles the effects we observed upon adding CHIR99021 to active NSCs (Fig. 5D-F). Remarkably, treating quiescent NSCs with 5 µM CHIR99021 resulted in increases in the proportions of EdU-positive and Ki67-positive cells compared with vehicle-treated controls (Fig. 6B,C). This contrasts with the marked decrease in proliferation observed with 5 µM CHIR99021 treatment of active NSCs (Fig. 5B,C). We also investigated neuronal differentiation and found that neither the expression levels of *Tuj1* nor the percentage of TUJ1+ cells were significantly changed in 5 µM CHIR99021-treated quiescent NSCs relative to vehicle-treated control cells (Fig. 6D-F). Overall, these data show that moderate stimulation of Wnt/β-catenin signalling promotes the activation of quiescent NSCs, whereas a higher level of stimulation promotes neuronal differentiation. This suggests that quiescent NSCs respond to Wnt/β-catenin signalling in a dose-dependent manner.

We next investigated whether the duration of CHIR99021 treatment influences the response of quiescent NSCs. To do so, we performed a time course study, in which quiescent NSCs were treated with 5 µM CHIR99021 for 24 h, 48 h and 72 h. We found that 5 µM CHIR99021 treatment induced a similar increase in *Axin2* expression levels across all time points (Fig. S7A), indicating a constant level of Wnt/β-catenin stimulation over time. The proportion of Ki67-positive cells increased with time in 5 µM CHIR99021-treated quiescent NSCs (Fig. S7B,C), whereas the expression of *Tuj1* was not significantly upregulated at any time point (Fig. S7D). Overall, this suggests that sustained 5 µM CHIR99021 treatment promotes the activation of an increasing proportion of quiescent NSCs but does not initiate their neuronal differentiation.

**Fig. 7.**
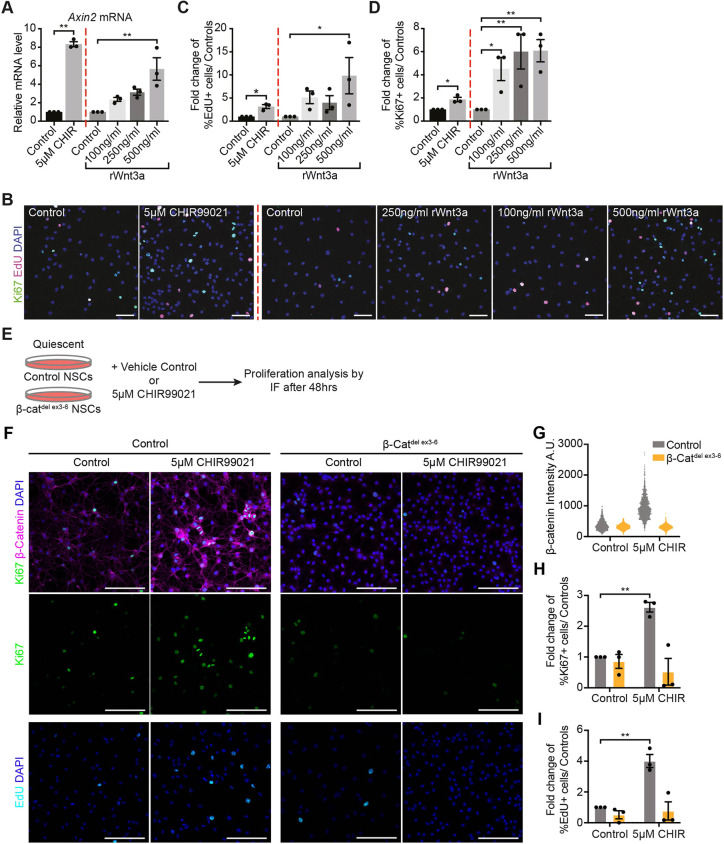
**Reactivation of quiescent NSCs by CHIR99021 treatment is dependent on intact Wnt/β-catenin signalling.** (A) *Axin2* expression in quiescent NSCs after 48 h of sustained 5 µM CHIR99021 and 500 ng/ml Wnt3a treatment. Results are shown normalised to the vehicle control for each Wnt/β-catenin agonist. *n*=3. (B) Immunolabelling of EdU, Ki67 and DAPI in quiescent NSCs treated with CHIR99021, rWnt3a and the vehicle controls for each agonist. (C,D) Quantification of the data shown in B: fold change in the proportion of EdU+ cells (C) and proportion of Ki67+ cells (D) for CHIR99021- and rWnt3a-treated quiescent NSCs are shown normalised to the vehicle control for each Wnt/β-catenin agonist. *n*=3. (E) Control and β-cat^del ex3-6^ quiescent NSCs were treated with either vehicle control or 5 µM CHIR99021 for 48 h before samples were collected for immunofluorescence analysis of proliferation markers. (F) Immunolabelling of β-catenin, Ki67, EdU and DAPI in control and β-cat^del ex3-6^ NSCs treated with either vehicle control or 5 µM CHIR99021 for 48 h. (G) Quantification of the experiment shown in F of the intensity of β-catenin staining in quiescent control and β-cat^del ex3-6^ NSCs following 5 µM CHIR99021 treatment. (H,I) Quantification of the fold change in the proportion of Ki67+ cells (H) and of EdU+ cells (I) in three independent experiments. *n*=3 (see also Fig. S8 and Materials and Methods). Data analysed with paired two-tailed Student's *t*-test for 5 µM CHIR99021 compared with control and repeated measures one-way ANOVA with Dunnett's multiple comparisons test for rWnt3a treatments compared with control (A,C,D), and two-way ANOVA with Sidak's multiple comparisons test (H,I): ns, *P*>0.05, *P<0.05, **P<0.01. Data are mean±s.e.m. Scale bars: 50 µm in B; 100 µm in F. A.U., arbitrary units.

We confirmed the results obtained with CHIR99021 by treating quiescent NSCs with recombinant Wnt3a (rWnt3a). *Axin2* was upregulated in a dose-dependent manner by different concentrations of rWnt3a, with 500 ng/ml rWnt3a inducing *Axin2* expression to a similar level as 5 µM CHIR99021 (Fig. 7A). rWnt3a also increased Ki67 expression and EdU incorporation in a dose-dependent manner (Fig. 7B-D). Additionally, we confirmed that the reactivation of quiescent NSCs induced by CHIR99021 stimulation is β-catenin dependent, because reactivation was fully abrogated in quiescent β-cat^del ex3-6^ NSCs treated with 5 µM CHIR99021 compared with control NSCs (Fig. 7E-I; Fig. S8). To further test the capability of β-catenin to promote NSC activation, we generated a NSC line from Catnb^lox(ex3)^ mice, induced quiescence and then stabilised β-catenin by the addition of Cre virus (or Null virus as control) (Fig. S9A). β-catenin was effectively stabilised following Cre transduction of Catnb^lox(ex3)^ NSCs, and the number of proliferating cells was higher in Catnb^del(ex3)^ NSCs compared with controls (Fig. S9). In addition, there was a slight increase in the proportion of TUJ1-positive cells (Fig. S9E), reminiscent of the increase in TUJ1-positive cells induced by 5 µM CHIR99021 treatment (Fig. 6F), suggesting that stabilising β-catenin in quiescent NSCs stimulates Wnt/β-catenin signalling similarly to 5 µM CHIR99021 treatment. Combined, these results confirm that directly stimulating Wnt/β-catenin signalling in quiescent NSCs promotes their activation.

## DISCUSSION

In this study, we used genetic and pharmacological approaches to stimulate and inhibit Wnt/β-catenin signalling directly in quiescent and active NSCs, both *in vivo* and *in vitro*. We found that Wnt/β-catenin signalling was low in the DG niche and that, whereas Wnt/β-catenin inhibition did not affect NSC homeostasis, Wnt/β-catenin stimulation exerted dose-dependent and state-specific effects on NSCs.

We found that Wnt/β-catenin signalling levels were low in NSC cultures and in the DG compared with the SVZ, and that deletion of β-catenin had no effect on Axin2 levels in NSCs *in vitro* or *in vivo*. Our finding that baseline Wnt/β-catenin signalling was not essential for the maintenance of the activation states or stem cell characteristics of NSCs differs from published reports showing that ablation of endogenous Wnt/β-catenin signalling affects NSC proliferation and multipotency (Wexler et al., 2009; Qu et al., 2013, 2010). Although we specifically targeted adult NSCs in our *in vivo* experiments, previous reports used knockout mouse models, making it difficult to distinguish between developmental and adult phenotypes and between cell-autonomous and non-cell-autonomous effects caused by the loss of Wnt/β-catenin signalling (Qu et al., 2010, 2013). In addition, although we used genetic ablation of β-catenin in NSC cultures, other groups used approaches based on the extracellular sequestration of Wnt ligands (such as Fzd-CRD), which could affect both Wnt/β-catenin and noncanonical Wnt signalling (Wexler et al., 2009; Qu et al., 2010). Noncanonical Wnt/PCP signalling maintains NSC quiescence in the V-SVZ but the role of Wnt/PCP in the SGZ has not yet been explored (Chavali et al., 2018). Our results corroborate work by Kuwabara et al. (2009) showing that loss of β-catenin in SOX2-positive proliferative progenitors *in vivo* does not affect the maintenance of the SOX2-positive progenitor pool. However, the authors used a retroviral approach to target proliferating cells uniquely and did not discriminate between the effects in active NSCs and intermediate progenitors (Kuwabara et al., 2009). By conditionally targeting the deletion of β-catenin to Glast-positive NSCs, we directly assessed the role of Wnt/β-catenin signalling specifically in NSCs, including quiescent NSCs. Our results showed that Wnt/β-catenin signalling was not required for NSC maintenance and function. We also found that loss of β-catenin in adult NSCs had no effect on the subsequent generation and proliferation of intermediate precursor cells or the generation of neuronally committed precursors. This contradicts previous work from several labs showing that neuronal differentiation is impaired following the loss of β-catenin signalling in proliferative progenitors or post-mitotic neuroblasts (Kuwabara et al., 2009; Karalay et al., 2011; Gao et al., 2007; Heppt et al., 2020). A possible explanation could be that Glast-dependent deletion occurs at a much earlier time point (when NSCs are still quiescent) compared with the acute loss of Wnt/β-catenin signalling performed in previous studies, therefore allowing more time for compensatory mechanisms. Of note, although we did not observe a difference in neurogenesis, we cannot exclude that β-catenin is needed for the functional maturation or integration of newly generated neurons.

Numerous reports show that stimulating Wnt/β-catenin signalling increases adult neurogenesis by promoting progenitor proliferation and the maturation of newborn neurons (Lie et al., 2005; Jang et al., 2013; Seib et al., 2013; Qu et al., 2010). Our finding that Wnt/β-catenin signalling is dispensable for adult neurogenesis does not exclude a regulatory role of Wnt/β-catenin stimulation on NSCs and their progeny. To stimulate Wnt/β-catenin signalling directly in NSCs *in vivo*, we first tried a genetic approach to stabilise β-catenin, which involved the deletion of exon 3, harbouring the GSK3β phosphorylation and α-catenin-binding sites (Harada et al., 1999). Upon recombination, we not only observed increased Wnt/β-catenin activity in NSCs, but also their displacement from the SGZ in Catnb^del(ex3)^ mice. Disruption of β-catenin binding to actin via α-catenin could affect the adhesion properties of recombined NSCs, contributing to the displacement phenotype we observed and to their subsequent loss. Indeed, disruption of β-catenin/cadherin signalling and loss of cell-cell contacts can disrupt tissue organisation and promote cell migration (Berx and Van Roy, 2001; Conacci-Sorrell et al., 2002; Taniguchi et al., 2006; Machon et al., 2007; Kadowaki et al., 2007). Interestingly, deletion of β-catenin using β-cat^del ex2-6^ mice (Brault et al., 2001) did not affect NSC positioning despite the potential of this mouse model to disrupt the function of β-catenin in cell adhesion in addition to transcriptional regulation. This could be because of the differences between inhibition of baseline Wnt/β-catenin signalling versus gain-of-function effects or a compensation from γ-catenin, which has been shown to be able to replace the function of β-catenin at adherens junctions in hepatocytes (Wickline et al., 2013). Nevertheless, the lack of phenotype suggests that neither of the functions of β-catenin in cell adhesion or transcriptional regulation are crucial for NSC homeostasis in the DG and precluded using other available conditional mice that would address the specific contribution of distinct β-catenin domains to its function (Draganova et al., 2015). The early appearance of the displacement phenotype in the Catnb^del(ex3)^ mice prevented us from using this mouse model to investigate the effects of Wnt/β-catenin stimulation on NSC behaviour. To overcome this problem, we used an *in vitro* model of hippocampal NSCs, which allows the culture of pure and homogeneous populations of NSCs in a controlled environment. We showed that stimulating Wnt/β-catenin signalling promotes neuronal differentiation of active NSCs while promoting the proliferation or differentiation of quiescent NSCs in a dose-dependent manner. The dose-dependent effects of stimulating Wnt/β-catenin signalling in quiescent NSCs shown here could provide a possible explanation for some of the contradictions reported in the literature, because different techniques used to modulate Wnt could stimulate Wnt/β-catenin signalling levels to varying degrees (Lie et al., 2005; Jang et al., 2013; Seib et al., 2013; Qu et al., 2010). For example, hippocampal-specific overexpression of Wnt ligands, which was found to promote neuronal differentiation (Lie et al., 2005), could induce a higher level of Wnt/β-catenin stimulation compared with knocking out a Wnt inhibitor, which was shown to enhance progenitor proliferation *in vivo* (Seib et al., 2013; Jang et al., 2013). These nontargeted approaches could induce secondary or non-cell-autonomous effects; therefore, novel *in vivo* tools will be needed to modulate and compare stimulated Wnt/β-catenin levels specifically in active and quiescent adult NSCs to confirm this hypothesis.

Dose-dependent effects of Wnt/β-catenin signalling have been reported in other systems, such as hematopoietic stem cells, intestinal stem cells and during cortical development and hippocampal cellular specification (Luis et al., 2011; Hirata et al., 2013; Machon et al., 2007). Specifically, low levels of Wnt/β-catenin stimulation preserve the self-renewal capacity of hematopoietic stem cells, whereas higher Wnt/β-catenin dosages enhance myeloid lineage differentiation (Luis et al., 2011). In NSCs, one potential mechanism mediating this dose-dependent response could be the dual regulation of *Neurod1* by SOX2 and the Wnt/β-catenin downstream effector TCF/LEF (Kuwabara et al., 2009). *Neurod1* harbours overlapping binding sites for both SOX2 and TCF/LEF, whereby SOX2 binding represses *Neurod1* expression in NSCs and Wnt/β-catenin signalling induces *Neurod1* expression in a dose-dependent manner (Kuwabara et al., 2009). The higher level of Wnt/β-catenin stimulation induced by 10 µM CHIR99021 could override the SOX2 repression of *Neurod1* in quiescent NSCs to induce neuronal differentiation, whereas the lower 5 µM CHIR99021 Wnt/β-catenin stimulation might not be sufficient. Although lower Wnt/β-catenin stimulation in quiescent NSCs promotes their activation, we also observed a mild, but not significant, upregulation of *Tuj1*. Further investigation is needed to determine whether this activation is linked to a loss of stemness and increased neuronal commitment or whether Wnt/β-catenin-activated NSCs are able to self-renew and return to a resting state of quiescence.

Only a small fraction of NSCs showed active Wnt signalling at any time point. We initially hypothesised that this might be linked to their activation status, but our results show that Wnt signalling is not correlated to NSC activation. The cause of the heterogeneous response of NSCs to Wnt remains to be determined, with possible contributors being oscillations in Wnt signalling, varying local levels of Wnt or the existence of a specific Wnt-responding NSC population.

Although our results showed that Wnt/β-catenin signalling is dispensable for NSC homeostasis in young adult mice, NSCs did still respond to Wnt/β-catenin stimulation, which could be important to mediate their response to external stimuli. For example, Wnt ligand levels increase following exercise in the DG of aged mice (Okamoto et al., 2011) and expression of the granule neuron-derived Wnt inhibitor SFRP3 is decreased by granule neuron activity (Jang et al., 2013), suggesting that the level of Wnt/β-catenin signalling in the DG is regulated by extrinsic factors. Decreased Wnt/β-catenin signalling levels with age contribute to age-related decline in adult hippocampal neurogenesis (Miranda et al., 2012; Seib et al., 2013; Okamoto et al., 2011) and Wnt-mediated adult hippocampal neurogenesis has been shown to be important for learning and memory (Jessberger et al., 2009). This suggests that Wnt/β-catenin signalling integration in NSCs could be an important regulator of external stimuli of adult hippocampal neurogenesis and hippocampal functions.

Our finding that quiescent and active NSCs were differentially responsive to Wnt/β-catenin signalling means that quiescent and active NSCs could coordinate their response to stimulated Wnt/β-catenin signalling *in vivo* to promote neurogenesis and NSC activation simultaneously. These state-specific responses to Wnt/β-catenin signalling could be driven by quiescent and active NSCs integrating different niche-derived signalling pathways. For example, Notch and BMP signalling is higher in quiescent NSCs compared with active NSCs, in which they maintain and promote quiescence (Lavado and Oliver, 2014; Ables et al., 2010; Imayoshi et al., 2010; Mira et al., 2010; Bonaguidi et al., 2008). Wnt/β-catenin signalling cooperatively regulates many developmental processes together with Notch and BMP signalling [reviewed by Muñoz Descalzo and Martinez Arias (2012) and Itasaki and Hoppler (2010)]. In addition, two recent papers highlighted the importance of β-catenin signalling dynamics in determining Wnt function during adult neurogenesis (Heppt et al., 2020; Rosenbloom et al., 2020). Therefore, Wnt/β-catenin signalling levels and/or dynamics modulated by external stimuli could play a key role in determining the response of quiescent NSCs to other niche cues.

In conclusion, our results show not only that Wnt/β-catenin signalling is dispensable for NSC homeostasis, but also that Wnt/β-catenin stimulation exerts dose-dependent and state-specific effects on NSCs that could contribute to the regulation of adult hippocampal neurogenesis in response to external stimuli. Further work is needed to understand the downstream molecular mechanisms by which levels of Wnt/β-catenin signalling induce differential effects across various cell types.

## MATERIALS AND METHODS

### Mouse models

All procedures involving animals and their husbandry were performed according to the guidelines of the Francis Crick Institute, national guidelines and laws. This study was approved by the Animal Ethics Committee and by the UK Home Office (PPL PB04755CC). Mice were housed in standard cages under a 12 h light/dark cycle with *ad libitum* access to food and water. All experimental mice were of mixed background. Founder mice were crossed with MF1 mice and then backcrossed to littermates of the F1 generation. *Glast-CreERT2* [*Slc1a3^tm(cre/ERT2)Mgoe^*] (Mori et al., 2006) mice were crossed with *Rosa26-floxed-stop-YFP* [*RYFP; Gt(ROSA)26Sor^tm1(EYFP)Cos^*] (Srinivas et al., 2001) mice to generate NSC-specific tamoxifen-inducible mice with a YFP recombination reporter. These mice were then crossed with the following experimental strains: β-cat^fl/fl ex3-6^ (*Ctnnb1^tm2Bir^*) (Huelsken et al., 2001), β-cat^fl/fl ex2-6^ (*Ctnnb1^tm2Kem^*) (Brault et al., 2001) and Catnb^lox(ex3)^ (*Ctnnb1^tm1Mmt^*) (Harada et al., 1999). *Glast-CreERT2; β-cat^fl/fl ex3-6^; RYFP* mice, *Glast-CreERT2; β-cat^fl/fl ex2-6^; RYFP* mice and *Glast-CreERT2; Catnb^lox(ex3)^; RYFP* mice were crossed with BATGAL Wnt/β-catenin reporter mice (Maretto et al., 2003) to introduce the *BATGAL* Wnt/β-catenin reporter allele.

Experimental groups included a combination of mice from different litters within each strain, and both males and females were used for all *in vivo* studies. Ages of animals used are detailed below or in the figure legends.

### Primary cell cultures

Adult hippocampal NSC cultures were derived as previously described (Blomfield et al., 2019). Briefly, 7-to-8-week-old mice were sacrificed and the DG dissected (previously described by Walker and Kempermann, 2014). For each new line, the two DGs of a single mouse of the desired genotype were dissociated using the Neural Tissue dissociation kit (P) (Milteny Biotec, 130-092-628). NSCs were amplified in nonadherent conditions as neurospheres in basal media [DMEM:F12+L-glutamine+sodium bicarbonate (Gibco, 11320)]+1 mg/ml KCl (Sigma, P5405)+2 mg/ml bovine serum albumin (BSA; Sigma, A9056)+1× Neurocult Supplement (Stem Cell Technologies, 05701)+1× penicillin/streptomycin (Thermo Fisher Scientific, 15140)+10 ng/ml FGF2 (Protech, 450-33)+20 ng/ml EGF (Protech, 315-09)+2 µg/ml heparin (Sigma, H3393) for at least two passages before dissociation to adherent cultures. NSCs were propagated in basal media [DMEM/F-12+Glutamax (Invitrogen 31331-093)]+1× N2 Supplement (R&D Systems, AR009)+1× penicillin/streptomycin+2 µg/ml laminin (Sigma, L2020)+5 µg/ml heparin+20 ng/ml FGF2. Neurospheres and adherent NSCs were incubated at 37°C, 5% CO_2_ and routinely tested for mycoplasma contamination.

### Tamoxifen administration

To induce activation of CreERT2 recombinase, 2-month-old *GlastCreERT2; β-cat^fl/fl ex3-6^; RYFP* mice were administered 2 mg (57-67 mg/kg) 4-hydroxy-tamoxifen (Sigma, H6278) by intraperitoneal injection at the same time each day for 5 consecutive days. Given the high numbers of deaths, and following veterinary advice, we changed to use tamoxifen (Sigma, T5648) for all other experiments. Two-month-old mice were administered 2 mg (57-67 mg/kg) tamoxifen by intraperitoneal (ip) injection at the same time each day for 5 consecutive days.

Approximately 2 weeks after tamoxifen administration, *Glast-CreERT2; Catnb^lox(ex3)^; RYFP* mice developed a skin phenotype resulting from the stabilisation of β-catenin in Glast-expressing activated hair follicle stem and progenitor cells (Reichenbach et al., 2018). We were able to collect a sufficient number of samples from this first cohort of mice to perform meaningful analysis 30 days after tamoxifen injection. However, all subsequent analyses were performed a maximum of 10 days after tamoxifen injection, following veterinary advice, before the skin phenotype developed.

### Tissue preparation and immunofluorescence

Mice were transcardially perfused, under terminal anaesthesia, with PBS for 2 min, followed by 4% paraformaldehyde (PFA) in PBS for 12 min. Brains were removed and post-fixed overnight in 4% PFA at 4°C with rocking and then transferred to PBS containing 0.02% sodium azide. Brains were coronally sectioned at a thickness of 40 µm using a vibratome (Leica).

Cultured NSCs were fixed with 4% PFA in PBS for 10 min at room temperature and then washed with PBS. Antigen retrieval was performed in brain sections prior to immunofluorescence using the following antibodies: GFP (Abcam, ab13970; 1:2000), β-catenin (BD Biosciences, 610154; 1:100), DCX (Santa Cruz, sc8066 discontinued; 1:50), Ki67 (BD Biosciences, 550609; 1:100), SOX2 (EBioscience, 14-9811-82; 1:400) and TBR2 (Abcam, ab183991; 1:200). For antigen retrieval, samples were incubated at 95°C for 2 min in sodium citrate buffer (10 mM, pH 6.0). Following antigen retrieval, samples were processed for immunofluorescence as previously described (Blomfield et al., 2019). Prior to mounting, brain sections were incubated with 2 µg/ml DAPI (Sigma, D9542) in 1:1 PBS:H_2_O for 30 min and fixed NSCs were incubated with 2 µg/ml DAPI in 1:1 PBS:H_2_O for 10 min at room temperature. Primary and secondary antibodies and dilutions are listed in Table S1. EdU was detected prior to DAPI incubation using the Click-iT™ EdU Alexa Fluor 647 detection kit (Invitrogen, C10340) following manufacturer's instructions.

### Microscopic analysis

Immunofluorescence samples were imaged using a SP5 confocal microscope (Leica) with a 40× oil objective lens (Leica). For fixed NSCs, three random regions of each coverslip were imaged with a 1 µm *z*-step. For each brain section, the left and right DGs in every 12th 40 µm section along the rostrocaudal axis were imaged, with a 1 µm *z*-step through the whole 40 µm section. For the reactivation experiment in quiescent control and β-cat^del ex3-6^ NSCs and quiescent Catnb^lox(ex3)/wt^ NSCs, an Axio Imager.Z2 microscope with a 20× objective (Zeiss) with Hamamatsu Orca Flash 4 camera and Apotome 2 technology for optical sectioning was used. Images of the comparison between DG and SVZ for immunolabelling of β-catenin were acquired with an Axio Observer microscope (Zeiss) equipped with a Yokogawa CSU X1 spinning disk and a Hamamatsu EMCCD camera.

### Cell treatments, constructs, transfection and transduction

To induce quiescence, cells were plated in flasks or on coverslips in active culture conditions and allowed to adhere overnight. The next day, media was replaced with basal media plus 20 ng/ml BMP4 (R&D Systems, 5020-BP) and incubated for 72 h at 37°C, 5% CO_2_ to establish quiescence as previously described (Blomfield et al., 2019). All experiments using quiescent NSCs were started following this 72 h incubation with BMP4 to ensure robust quiescence induction.

To label cells in S-phase of the cell cycle, EdU (Invitrogen, C10340) was added to the media of cultured cells at a final concentration of 10 µM 1 h before fixation.

To delete β-catenin in NSCs derived from *β-cat^fl/fl ex3-6^; RYFP* mice, active and quiescent β-cat^fl/fl ex3-6^ NSCs were transduced with either adenovirus control (Ad-CMV-Null, Vector Biolabs, 1300) or adenovirus expressing Cre (Ad-CMV-iCre, Vector Biolabs, 1045) at a multiplicity of infection (MOI) of 100. Samples of active β-cat^fl/fl ex3-6^ NSCs were collected 1, 2 and 3 days after transduction for quantitative (q)PCR, immunofluorescence and western blot analyses. Samples of quiescent β-cat^fl/fl ex3-6^ NSCs were collected 2, 4 and 6 days after transduction for qPCR and immunofluorescence analyses. To stimulate Wnt/β-catenin signalling in active β-cat^fl/fl ex3-6^ NSCs, media was replaced with basal media plus 5 µM CHIR99021 (BioVision, 1677-5) or DMSO vehicle control 48 h after transduction and incubated for 24 h at 37°C, 5% CO_2_ before collection in TRIzol (Ambion, 15596018) for RNA extraction, cDNA synthesis and qPCR analysis. To determine the reactivation rate of quiescent β-cat^fl/fl ex3-6^ NSCs, media was replaced with basal media 48 h after transduction and samples were collected 3 and 6 days later for immunofluorescence analysis. Samples were also collected in basal media plus 20 ng/ml BMP4 48 h after transduction for the 0 h time point. Media was refreshed every 72 h. To determine how chronic loss of β-catenin affects neurogenesis and gliogenesis, the media on transduced active β-cat^fl/fl ex3-6^ NSCs was replaced at 6, 12 and 18 days after transduction with basal media minus 20 ng/ml FGF2 plus 2% B27 to promote neuronal differentiation and basal media minus 20 ng/ml FGF2 plus 2% FBS to promote astrocytic differentiation. Cells were incubated in these differentiation media conditions for 5 days at 37°C, 5% CO_2_ before fixation. To confirm that 5 µM CHIR99021-induced reactivation of quiescent NSCs was dependent upon Wnt/β-catenin signalling, quiescent control and β-cat^del ex3-6^ NSCs were treated with either DMSO vehicle control or 5 µM CHIR99021 for 48 h before fixation for immunofluorescence analysis. Of note, we observed that treatment with CHIR99021 immediately after viral transduction often resulted in poor viability of the cultures. Therefore, we performed this experiment using two alternative protocols, with the viral transduction step either in proliferating or already-quiescent NSCs (Fig. S8).

To stimulate Wnt/β-catenin signalling in active NSCs, media on cells was replaced with basal media supplemented with DMSO vehicle control, 1 µM CHIR99021, 5 µM CHIR99021 or 10 µM CHIR99021 and incubated for 48 h at 37°C, 5% CO_2_ before sample collection. To stimulate Wnt/β-catenin signalling in quiescent NSCs, media on cells was replaced with basal media plus 20 ng/ml BMP4 supplemented with DMSO vehicle control, 1 µM CHIR99021, 5 µM CHIR99021 or 10 µM CHIR99021 and incubated for 24 h, 48 h and 72 h at 37°C, 5% CO_2_ before sample collection. To compare the effects of CHIR99021 treatment versus rWnt3a (R&D Systems, 1324-WN) in quiescent NSCs, media on cells was replaced with basal media plus 20 ng/ml BMP4 supplemented with DMSO (vehicle control for CHIR99021), 5 µM CHIR99021, 0.1% BSA/PBS (vehicle control for rWnt3a), 100 ng/ml rWnt3a, 250 ng/ml rWnt3a or 500 ng/ml rWnt3a and incubated 48 h at 37°C, 5% CO_2_ before sample collection. Media was refreshed every 24 h. To stabilise β-catenin in NSCs derived from *Catnb^lox(ex3)^; RYFP* mice, quiescent Catnb^lox(ex3)/wt^ NSCs were transduced with either Ad-CMV-Null or Ad-CMV-iCre at a MOI=100. Control and Catnb^del(ex3)^ NSCs were collected 2 days after virus transduction for immunofluorescence analysis.

### Protein purification and western blots

For western blot analysis, samples were washed with ice-cold PBS and scraped in lysis buffer (Thermo Fisher Scientific, 87788) supplemented with a phosphatase inhibitor (Thermo Fisher Scientific, 78420), EDTA (Thermo Fisher Scientific, 87786) and a protease inhibitor (Thermo Fisher Scientific, 87786). Cells were lysed at 4°C for 20 min under rotation and then centrifuged at 13,000 rpm (16,200 ***g***) at 4°C for 20 min. The supernatant was transferred to a clean Eppendorf tube and an equal volume of Laemmli sample buffer (Sigma, S3401-10VL) was added before boiling at 95°C for 5 min.

Samples were run on a polyacrylamide gel at 120 V and then transferred onto a nitrocellulose membrane. Membranes were blocked with 5% milk in 0.1% TBS-Tween (Bio-Rad, 1706435; Sigma-Aldrich, P9416) or 5% BSA in TBS-Tween before incubation with primary antibody diluted in 5% milk in TBS-Tween (actin, β-catenin and vinculin) or 5% BSA in TBS-Tween+0.02% sodium azide (Wnt7a) overnight at 4°C under rotation. The following day, membranes were washed with TBS-Tween and incubated with secondary antibody diluted in 5% milk in TBS-Tween for 2 h at room temperature with rocking. Detection was performed using ECL Western Blotting reagents (Sigma, GERPN2106) according to the manufacturer's instructions and blots were developed using Kodak films. Primary and secondary antibodies and dilutions are listed in Table S1.

### Dentate gyrus dissections and fluorescence activated cell sorting

Two-month-old control, β-cat^del ex3-6^ and β-cat^del ex2-6^ mice were injected with tamoxifen for 5 consecutive days and culled 10 days later. The DG was dissected as previously described (Walker and Kempermann, 2014). Dissected DGs were dissociated using the Neural Tissue dissociation kit (P) (Milteny Biotec, 130-092-628) as previously described (Harris et al., 2021). Dissociated cells were centrifuged and resuspended in 750 µl recovery media [0.5% PBS/BSA in DMEM/F12 without Phenol Red (Sigma, DG434) and 1 µg/ml DAPI]. Cells were sorted on a MoFLo XDP cell sorter machine (Beckman Coulter) using a 100 µm nozzle at a maximum sort speed of 5000 events per second and an efficiency of more than 80%. Events were first gated to remove debris (fsc-h versus ssc-h), aggregates (pulse width versus area then pulse height versus area/width) and dead cells (fsc-h versus DAPI fluorescence). Cells were then gated for YFP expression using a YFP-negative control mouse. Mice were processed for FAC sorting (FACS) separately and YFP-positive cells from individual samples were collected directly in 700 µl RL lysis buffer from the RNeasy^®^ Mini Kit (Qiagen, 74104) for RNA extraction.

### RNA extraction, cDNA synthesis and qPCR

For FACS experiments, cells were lysed using the RL lysis buffer from the RNeasy Mini Kit. For all other experiments, cells were lysed using TRIzol reagent (Ambion, 15596018). RNA was extracted using the RNeasy Mini Kit or the Direct-zol RNA MiniPrep Kit (Zymo Research, R2052) according to the manufacturers’ instructions. cDNA was synthesised using the Maxima First Strand cDNA Synthesis Kit (Thermo Fisher Scientific, K1641) or the High Capacity cDNA Reverse Transcription Kit (Applied Biosystems, 4387406) according to the manufacturers’ instructions. Gene expression levels were measured using TaqMan Gene expression assays (Applied Biosystems, Table S2) and qPCR was performed on a QuantStudio Real-Time PCR machine (Thermo Fisher Scientific). Gene expression was calculated relative to the endogenous controls *GAPDH* and *ActinB* (dCt) and to the control samples to provide a delta delta cycle threshold (ddCt) value. ddCT values were then converted to the fold change in gene expression relative to control. All samples were measured in technical duplicates for each qPCR run and then averaged.

### RNA-sequencing and analysis

The publicly available single cell RNA-sequencing dataset of *in vivo* NSCs (Harris et al., 2021) was accessed from GEO (GSE159768) with associated code (https://github.com/harrislachlan/lifelong_stemcells). The final dataset contained 2947 NSCs, which were classified as dormant, resting and active NSCs as described by Harris et al. (2021). For this study, the metadata were appended to classify dormant and resting NSCs as quiescent NSCs. Active NSC classification remained the same. Differential gene expression analysis was performed using the FindMarkers function in Seurat (Version 3.1.1) (Stuart et al., 2019) using the Pearson residuals located in the ‘scale.data’ slot of the SCT assay using the Student's *t*-test (Hafemeister and Satija, 2019). All genes that were expressed by a minimum of 10% of cells were tested for differential expression. No minimum log fold change threshold was enforced. The heatmap in Fig. 1A was generated using the DoHeatmap function in Seurat. The publicly available bulk RNA-sequencing of active and quiescent NSC cultures (Blomfield et al., 2019) was accessed from GEO (GSE116997).

### Quantifications and statistical analysis

#### Immunofluorescence quantifications

NSCs in DG images from BATGAL mice were identified as having a DAPI-positive cell body located within the SGZ colocalised with a GFAP-positive radial projection through the GCL spanning a minimum of two cell nuclei away from the NSC nucleus. NSCs in all other DG images were identified not only as above, but also as being YFP positive and having a SOX2-positive cell body. Displaced NSCs were identified as above but with the DAPI-positive SOX2-positive cell body being located a distance of more than two nuclei away from the SGZ. To measure the intensity of BATGAL and Id4 immunofluorescence staining, the nucleus of each BATGAL-positive or Id4-positive NSC was manually outlined according to DAPI, and the mean pixel value of the channel of interest was measured using Fiji software. Each value was normalised to the average BATGAL or Id4 intensity level measured in DAPI-positive cells in the GCL from the same *z*-plane as each NSC. To quantify the proportion of cells expressing specific markers in DG images, a minimum of 100 cells were counted per animal for a minimum of three animals per genotype. To quantify the total number of cells per mm length of SGZ, a minimum of two sections per series per mouse was quantified for a minimum of three mice per genotype. The freehand line tool in Fiji (Schindelin et al., 2012) was used to measure the length of the SGZ in µm and was converted to mm for analysis. All samples were blinded to the experimenter prior to quantification.

To quantify cultured immunolabelled NSC samples, the DAPI channel was used to generate a mask of all nuclei using Fiji software. This mask was used to measure the average pixel intensity for each channel within the area of each nucleus. Cytoplasmic quantifications (e.g. for TUJ1) were made using a ring mask around each nucleus. The proportion of cultured NSCs expressing specific markers was quantified by setting a signal threshold and expressing the number of positive cells as a percentage of the total number of cells. For each experiment, at least 100 cells were quantified across three biological replicates unless stated otherwise.

#### Statistical analysis

Determination of the appropriate sample sizes (*n*) was based on experiments from our previous publications (Andersen et al., 2014; Blomfield et al., 2019; Urbán et al., 2016; Harris et al., 2021) and similar published reports from other groups. All animals were included in the final analysis.

All statistical analyses were performed using the GraphPad Prism 7 (Version 7.0c) or GraphPad Prism 9 software (Version 9.2.0. RRID:SCR_002798). Appropriate statistical tests for each type of data were used as follows: a two-tailed unpaired Student's *t*-test; two-tailed paired Student's *t*-test; ordinary one-way ANOVA with Tukey's multiple comparison test; repeated-measures one-way ANOVA with Dunnett's multiple comparison test and two-way ANOVA with Sidak's or Tukey's multiple comparisons tests as appropriate. All error bars represent the mean±s.e.m. Significance is stated as follows: ns, *P*>0.05; *P<0.05; **P<0.01; ***P<0.001. The statistical details and sample sizes for each experiment are recorded in the figure legends. Sample sizes in the figure legends represent biological replicates unless stated otherwise.

## Supplementary Material

Supplementary information

Reviewer comments
