# Supplementary material for: Wnt/β-catenin signalling is dispensable for adult neural stem cell homeostasis and activation
Source: Development. 2021 Oct 19;148(20):dev199629. doi: 10.1242/dev.199629 (PMC8572000; doi:10.1242/dev.199629)
Supplement: Supplementary information [file develop-148-199629-s1.pdf]

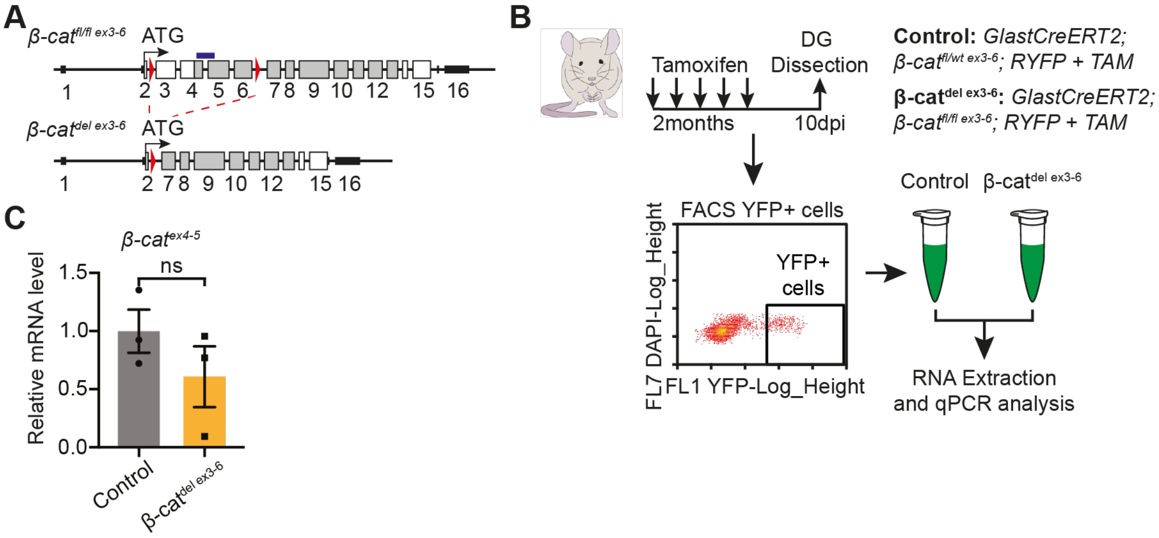

Fig. S1. Analysis of failed *β-catenin* recombination in β-cat$^{del\ ex3\text{-}6}$ mice.

(**A**) *β-cat*$^{fl/fl\ ex3\text{-}6}$ allele before and after recombination. LoxP sites (red arrows) flank exons 3-6, which encode the N-terminal domain and armadillo repeats 1-4. Grey boxes indicate armadillo repeats. Blue box indicates the sequence recognised by the TaqMan probe used for qPCR analysis in (**C**).

(**B**) Two-month-old Control and β-cat$^{del\ ex3\text{-}6}$ mice were injected with tamoxifen once daily for 5 consecutive days to induce recombination of the *β-cat*$^{fl/fl\ ex3\text{-}6}$ allele. Ten days after the first tamoxifen injection, the DG of individual mice were dissected and YFP+ cells were collected by flow cytometry for RNA extraction and qPCR analysis.

(**C**) *β-cat*$^{ex4\text{-}5}$ (blue box, **A**) expression is not significantly different between YFP+ cells collected by flow cytometry from the DG of Control and β-cat$^{del\ ex3\text{-}6}$ mice indicating failed recombination. n=3. Statistics: unpaired two-tailed Student's t-test. (ns, $p>0.05$). Error bars represent mean with SEM.

Development • Supplementary information

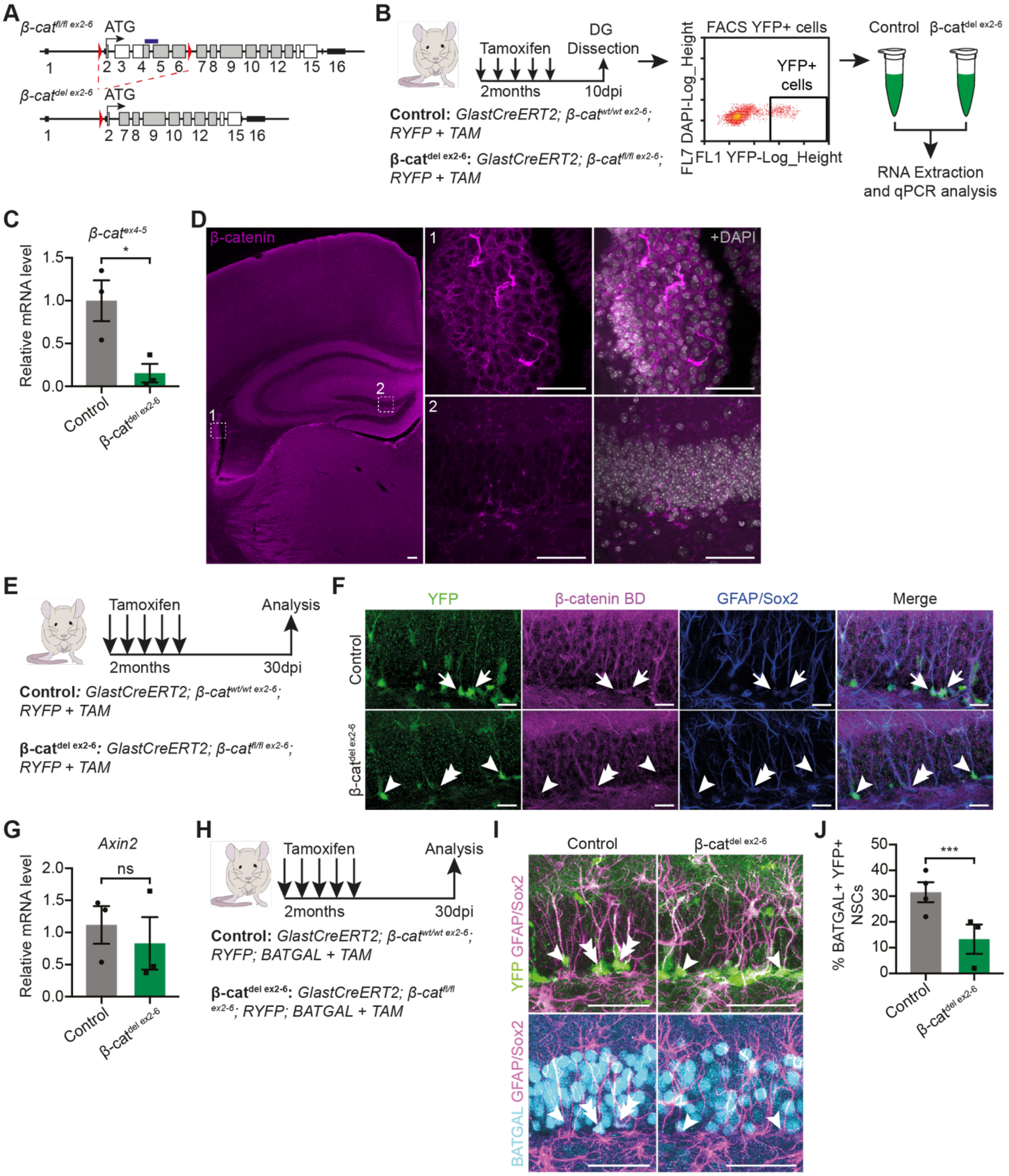

Development • Supplementary information

Fig. S2. Analysis of successful *β-catenin* recombination in β-cat$^{del\ ex2\text{-}6}$ mice and resulting inhibition of Wnt/β-catenin signalling in β-cat$^{del\ ex2\text{-}6}$ mice.

(**A**) *β-cat*$^{fl/fl\ ex2\text{-}6}$ allele before and after recombination.

(B) Two-month-old Control and β-cat$^{del\ ex3\text{-}6}$ mice were injected with tamoxifen once daily for 5 consecutive days. Ten days after the first tamoxifen injection, the DG of individual mice were dissected and YFP+ cells were collected by flow cytometry for RNA extraction and qPCR analysis.

(**C**) *β-cat*$^{ex4\text{-}5}$ (blue box, **D**) expression is significantly decreased in β-cat$^{del\ ex2\text{-}6}$ YFP+ cells compared with Control YFP+ cells indicating successful *β-cat*$^{fl/fl\ ex2\text{-}6}$ recombination. n=3.

(**D**) Comparison of β-catenin immunolabelling in the DG and SVZ of a wild type two-month-old mouse. β-catenin immunolabelling marks the cellular membrane and is stronger in the SVZ compared with the DG. Scale bar, 50μm.

(**E**) Two-month-old Control and β-cat$^{del\ ex2\text{-}6}$ mice were administered tamoxifen for 5 consecutive days and sacrificed 30 days after the first tamoxifen injection.

(**F**) β-catenin, YFP, GFAP and Sox2 immunolabelling in the DG of Control and β-cat$^{del\ ex2\text{-}6}$ mice. Arrows in control indicate YFP-positive, β-catenin-positive NSCs. Arrowheads in β-cat$^{del\ ex2\text{-}6}$ mice indicate recombined YFP-positive, β-catenin-negative NSCs whereas the double arrowhead indicates an un-recombined YFP-negative, β-catenin-positive NSC. Scale bar, 50μm.

(**G**) *Axin2* expression is unchanged in β-cat$^{del\ ex2\text{-}6}$ YFP+ cells compared with Control YFP+ cells following FAC Sorting of YFP-positive cells in control and β-cat$^{del\ ex3\text{-}6}$ mice as described in (**B**). n=3.

(**H**) Two-month-old Control and β-cat$^{del\ ex2\text{-}6}$ mice, crossed with BATGAL Wnt/β-catenin reporter mice, were administered tamoxifen for 5 consecutive days and sacrificed 30 days after the first tamoxifen injection.

(**I**) BATGAL (β-galactosidase), GFAP and Sox2 immunolabelling in the DG of Control and β-cat$^{del\ ex2\text{-}6}$ mice crossed with *BATGAL* mice. Arrowheads indicate BATGAL- NSCs and double arrowheads indicate BATGAL+ NSCs. Scale bar, 50μm.

(**J**) Quantification of the proportion of BATGAL+ NSCs in **(H)**. The decreased proportion of BATGAL+ NSCs in β-cat$^{del\ ex2\text{-}6}$ mice (13.33 ± 5.69%, n=3) compared with Control (31.5 ± 3.88%, n=4) indicates successful inhibition of Wnt/β-catenin signalling following recombination of the β-cat$^{fl/fl\ ex2\text{-}6}$ allele.

Statistics: unpaired two-tailed Student's t-test (**C**, **G** and **J**). (ns, p>0.05. *, p<0.05. ***, p<0.001). Error bars represent mean with SEM.

Development • Supplementary information

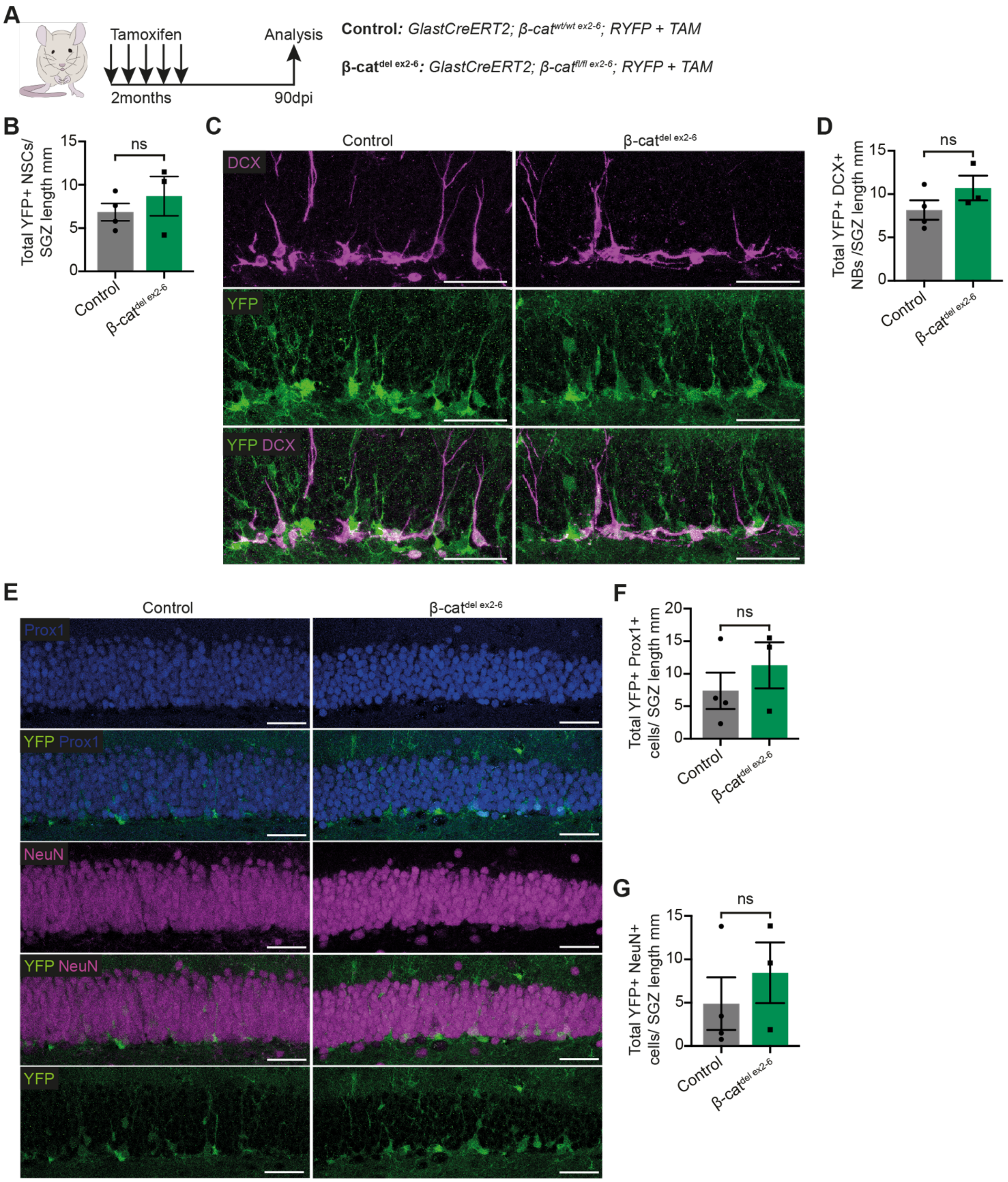

A
Tamoxifen
Analysis
2months
90dpi
Control: GlastCreERT2; β-cat wt/wt ex2-6; RYFP + TAM
β-cat del ex2-6: GlastCreERT2; β-cat fl/fl ex2-6; RYFP + TAM
B
Total YFP+ NSCs/ SGZ length mm
ns
Control
β-cat del ex2-6
C
Control
β-cat del ex2-6
DCX
YFP
YFP DCX
D
Total YFP+ DCX+ NBs /SGZ length mm
ns
Control
β-cat del ex2-6
E
Control
β-cat del ex2-6
Prox1
YFP Prox1
NeuN
YFP NeuN
YFP
F
Total YFP+ Prox1+ cells/ SGZ length mm
ns
Control
β-cat del ex2-6
G
Total YFP+ NeuN+ cells/ SGZ length mm
ns
Control
β-cat del ex2-6

Fig. S3. NSCs and adult hippocampal neurogenesis are unaffected by the long-term deletion of β-catenin and inhibition of Wnt/β-catenin signalling in NSCs.

(**A**) Two-month-old Control and β-cat$^{del\ ex2\text{-}6}$ mice were administered tamoxifen for 5 consecutive days and sacrificed 90 days after the first tamoxifen injection.

(**B**) Quantifications of the total number of NSCs (YFP+ GFAP+ Sox2+ radial cells in the SGZ) normalised to the length of the SGZ (mm) in Control (6.85 ± 1.00) and β-cat$^{del\ ex2\text{-}6}$ (8.69 ± 2.27) mice 90 days after tamoxifen administration. n=3

(**C**) YFP and DCX immunolabelling in the DG of Control and β-cat$^{del\ ex2\text{-}6}$ mice 90 days after tamoxifen administration. Scale bars, 50µm.

(**D**) Quantifications of the data shown in (**C**). The total number of neuroblasts (NBs, DCX+ YFP+ cells) normalised to the SGZ length (mm) are unchanged between Control (8.17 ± 1.12) and β-cat$^{del\ ex2\text{-}6}$ mice (10.72 ± 1.43), indicating that NBs are unaffected by long-term β-catenin deletion. n=3.

(**E**) YFP, Prox1 and NeuN immunolabelling in the DG of Control and β-cat$^{del\ ex2\text{-}6}$ mice 90 days after tamoxifen administration. Scale bars, 50µm.

(**F, G**) Quantifications of the data shown in (**C**). The total number of YFP+ Prox1+ cells normalised to the SGZ length (mm) (Control vs β-cat$^{del\ ex2\text{-}6}$: 7.38 ± 2.8 vs 11.3 ± 3.54, **F**) and the total number of YFP+ NeuN+ cells normalised to the SGZ length (mm) (Control vs β-cat$^{del\ ex2\text{-}6}$: 4.89 ± 3.02 vs 8.45 ± 3.5, **G**) are unchanged between Control and β-cat$^{del\ ex2\text{-}6}$ mice indicating that neurogenesis is unaffected by long-term β-catenin deletion. n=3.

Statistics: unpaired two-tailed Student's t-test (**B, D, F, G**). (ns, $p>0.05$). Error bars represent mean with SEM.

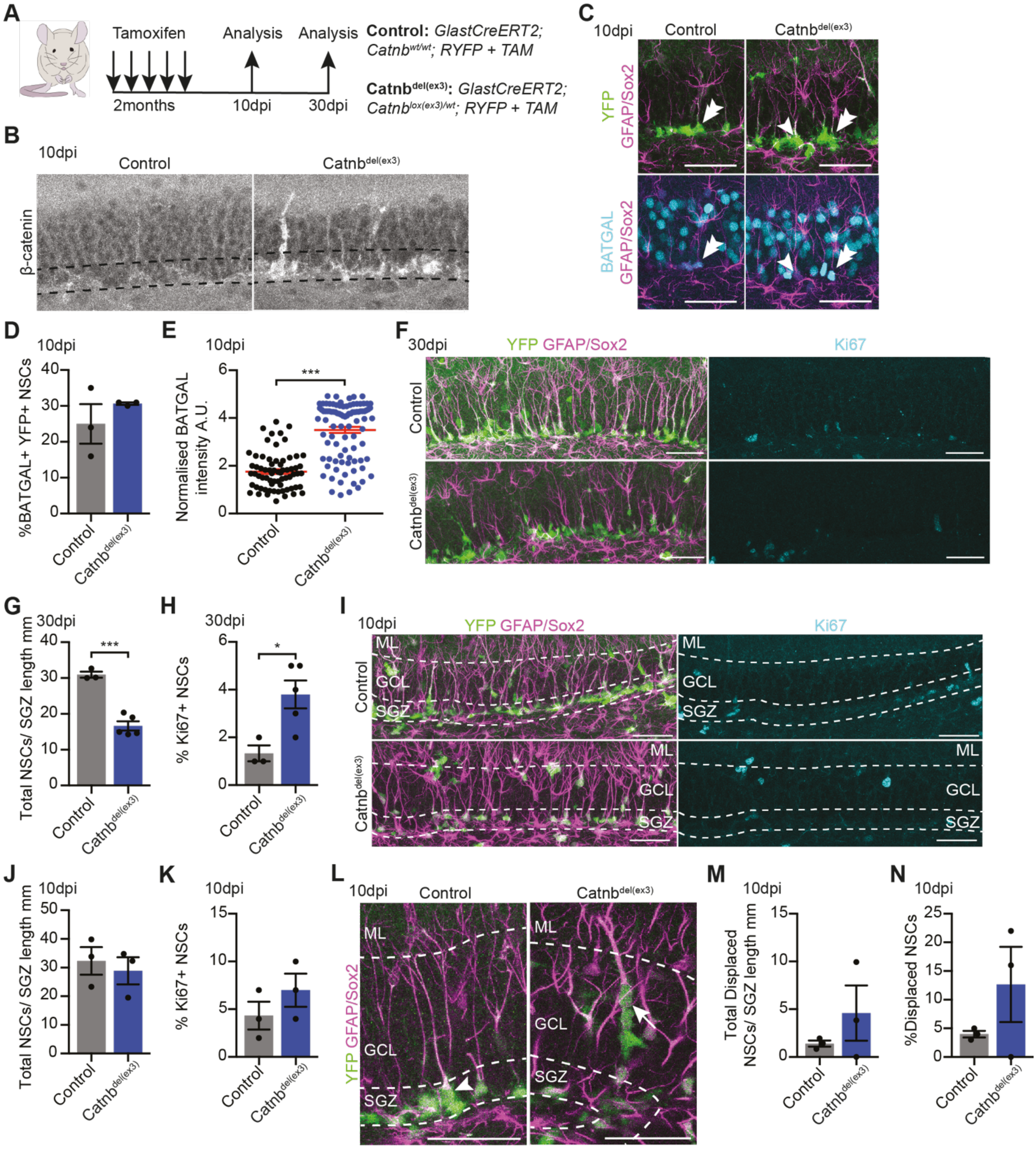

Development • Supplementary information

Fig. S4. Stabilising β-catenin in NSCs *in vivo* displaces them from their correct niche location.

(**A**) Two-month-old Control and $Catnb^{del(ex3)}$ mice were administered tamoxifen for 5 consecutive days and sacrificed 10 and 30 days after the first tamoxifen injection.

(**B**) β-catenin immunolabelling in the DG of Control and $Catnb^{del(ex3)}$ mice 10 days after tamoxifen. Increased β-catenin staining in $Catnb^{del(ex3)}$ mice indicates successful recombination. Dashed lines mark the SGZ. Scale bars, 50μm.

(**C**) BATGAL (β-galactosidase), GFAP and Sox2 immunolabelling in the DG of Control and $Catnb^{del(ex3)}$ mice crossed with BATGAL mice, 10 days after tamoxifen. Arrowheads indicate BATGAL- NSCs and double arrowheads indicate BATGAL+ NSCs. Scale bar, 50μm.

(**D**) Quantification of the proportion of BATGAL+ NSCs in (**C**). The proportion of BATGAL+ NSCs is unchanged between Control (25 ± 5.51%) and $Catnb^{del(ex3)}$ (30.67 ± 0.33%) 10 days after tamoxifen, which corresponds to the proportion of NSCs expressing *β-catenin* by RNA sequencing (32% quiescent NSCs. n=3.

(**E**) Quantification of the data shown in (**C**) of the intensity of BATGAL staining in BATGAL+ NSCs normalised to the BATGAL intensity in neighbouring DAPI+ GCL cells. BATGAL reporter intensity is increased in BATGAL+ NSCs in *Catnb*$^{del(ex3)}$ mice ($p<0.0001$). A.U.= Arbitrary Units. n=3

(**F**) YFP, GFAP, Sox2 and Ki67 immunolabelling in the DG of Control and $Catnb^{del(ex3)}$ mice 30 days after tamoxifen administration. Scale bars, 50μm.

(**G, H**) Quantifications of the data shown in (**F**) of the total number of NSCs normalised to SGZ length (mm) (Control vs $Catnb^{del(ex3)}$: 30.95 ± 0.86 vs 16.65 ± 1.26, **G**) and the proportion of Ki67+ NSCs (Control vs $Catnb^{del(ex3)}$: 1.33 ± 0.33% vs 3.8 ± 0.58%, **H**). NSCs are lost from the DG 30 after stabilising β-catenin and their proliferation is increased. n=3 for Control, n=5 for $Catnb^{del(ex3)}$.

(**I**) YFP, GFAP, Sox2 and Ki67 immunolabelling in the DG of Control and $Catnb^{del(ex3)}$ mice 10 days after tamoxifen administration. Scale bars, 50μm.

(**J, K**) Quantifications of the data shown in (**I**) of the total number of NSCs normalised to the length of the SGZ (mm) (Control vs $Catnb^{del(ex3)}$: 32.38 ± 4.83 vs 28.92 ± 4.72, **J**) and the proportion of Ki67+ NSCs (Control vs $Catnb^{del(ex3)}$: 4.33 ± 1.45% vs 7 ± 1.73%, **K**). Neither the total number of NSCs nor their proliferation are affected 10 days after stabilising β-catenin. n=3.

Development • Supplementary information

(L) Representative images of SGZ located (arrowheads) and displaced (arrows) NSCs (YFP+ GFAP+ Sox2+ NSCs) in the DG of Control and Catnbdel(ex3) mice 10 days after tamoxifen administration. Dashed lines mark the SGZ. Scale bars, 50µm.

(M, N) Quantifications of the data shown in (L). SGZ located NSCs (arrowheads) retain their correct niche location with their cell body located in the SGZ and a radial process through the GCL. Displaced NSCs in Catnbdel(ex3) mice (arrows) were identified as YFP+ GFAP+ Sox2+ NSCs that are located more than 2 cell nuclei away from the SGZ. The total number of displaced NSCs normalised to the SGZ length (mm) (Control vs Catnbdel(ex3): 1.42 ± 0.32 vs 4.59 ± 2.89, M) and the proportion of displaced NSCs (Control vs Catnbdel(ex3): 4 ± 0.58% vs 12.67 ± 6.57%, N). Displacement of NSCs is increased in Catnbdel(ex3) mice compared with Control. n=3.

SGZ, subgranular zone. GCL, granule cell layer. ML, molecular layer.

Statistics: unpaired two-tailed Student's t-test (**D**, **E**, **G**, **H**, **J**, **K**, **M** and **N**). (ns, $p>0.05$. *, $p<0.05$. ***, $p<0.001$). Error bars represent mean with SEM.

Development • Supplementary information

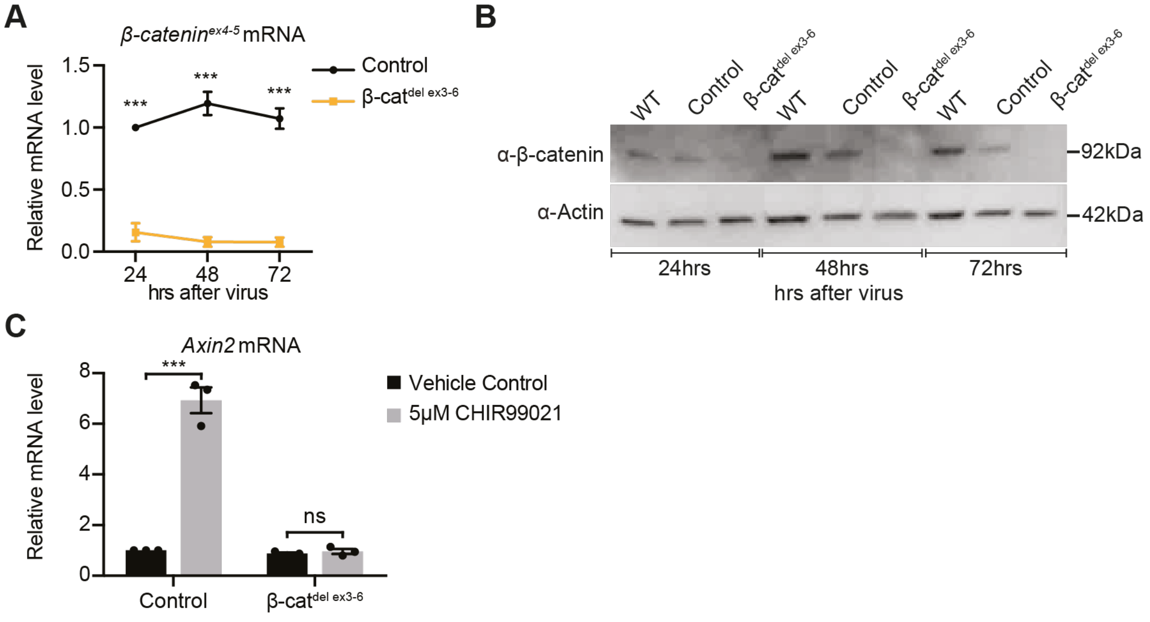

Fig. S5. Validation of β-catenin recombination and deletion in β-catdel ex3-6 NSCs.

(**A**) qPCR gene expression analysis of *β-cat* $^{ex4\text{-}5}$ (blue box, Figure S1**A**) 24hrs, 48hrs and 72hrs after control- and Cre-adenovirus transduction in Control and β-cat$^{del\ ex3\text{-}6}$ active NSCs. *β-cat* $^{ex4\text{-}5}$ lies within the floxed region of the *β-cat*$^{fl/fl\ ex3\text{-}6}$ allele and is downregulated in active β-cat$^{del\ ex3\text{-}6}$ NSCs vs Control ($p<0.0001$ for all time points). n=3.

(**B**) Western blot of β-catenin protein levels in wild type (WT, no virus), Control (control-adenovirus) and β-cat$^{del\ ex3\text{-}6}$ (Cre-adenovirus) active NSCs 24hrs, 48hrs and 72hrs after adenovirus transduction. β-catenin protein levels are abolished 48hrs after adenovirus transduction. n=3.

(**C**) β-cat$^{del\ ex3\text{-}6}$ active NSCs fail to upregulate *Axin2* in response to 5µM CHIR99021 treatment, indicating their inability to respond to a Wnt/β-catenin stimulus. CHIR99021 treatment began 48hrs after adenovirus transduction and maintained for 48hrs. n=3.

Statistics: Two-way ANOVA with Sidak's multiple comparisons test (**A**, **C**). (ns, $p>0.05$. ***, $p<0.001$). Error bars represent mean with SEM.

Development • Supplementary information

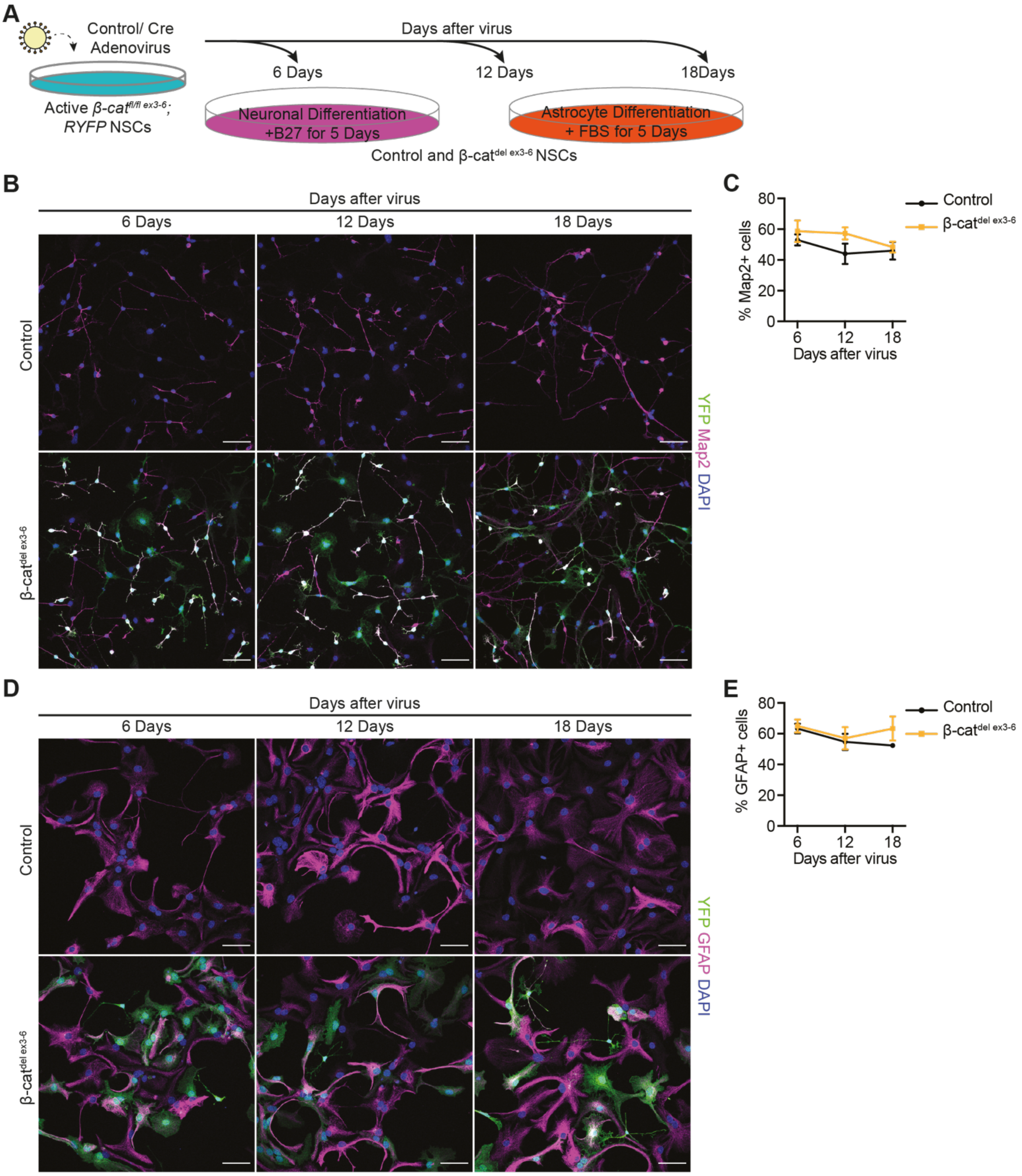
A
Control/ Cre Adenovirus
Active β-cat fl/fl ex3-6; RYFP NSCs
Days after virus
6 Days
12 Days
18Days
Neuronal Differentiation +B27 for 5 Days
Astrocyte Differentiation + FBS for 5 Days
Control and β-cat del ex3-6 NSCs
B
Days after virus
6 Days
12 Days
18 Days
Control
β-cat del ex3-6
YFP Map2 DAPI
C
% Map2+ cells
Days after virus
Control
β-cat del ex3-6
D
Days after virus
6 Days
12 Days
18 Days
Control
β-cat del ex3-6
YFP GFAP DAPI
E
% GFAP+ cells
Days after virus
Control
β-cat del ex3-6

Fig. S6. Chronic loss of Wnt/β-catenin signalling does not impair neuronal or astrocytic differentiation *in vitro*.

(**A**) Six-, 12- and 18-days after virus transduction Control and β-cat$^{fl/fl\ ex3\text{-}6}$ NSCs were cultured for 5 days in conditions to promote neuronal (+B27) and astrocytic (+FBS) differentiation.

(**B**) YFP, Map2 and DAPI immunolabelling in Control and β-cat$^{del\ ex3\text{-}6}$ NSCs cultured in B27 for 5 days at 6-, 12- and 18-days after virus transduction. YFP labels recombined cells and Map2 labels differentiated neurons. Scale bars, 50μm.

(**C**) Quantification of the proportion of Map2+ neurons in (**B**) (Control vs β-cat$^{del\ ex3\text{-}6}$: 6days = 53 ± 3.61% vs 58.67 ± 6.96%, 12days = 44 ± 6.66% vs 57.33 ± 3.93%, 18days = 46 ± 5.69% vs 48.33 ± 3.53%). Control and β-cat$^{del\ ex3\text{-}6}$ NSCs are similarly able to differentiate into neurons. Data shown are technical replicates of n=1 with an average of 147 cells counted per sample.

(**D**) YFP, GFAP and DAPI immunolabelling in Control and β-cat$^{del\ ex3\text{-}6}$ NSCs cultured in FBS for 5 days following 6-, 12- and 18-days after virus transduction. YFP labels recombined cells and GFAP labels differentiated astrocytes. Scale bars, 50μm.

(**E**) Quantification of the proportion of GFAP+ astrocytes in (**D**) (Control vs β-cat$^{del\ ex3\text{-}6}$: 6days = 63.33 ± 3.18% vs 65 ± 4.16%, 12days = 54.67 ± 5.18% vs 57 ± 7.21%, 18days = 52.33 ± 0.67% vs 63.33 ± 7.79%). Astrocytic differentiation is unaffected by loss of β-catenin. Data shown are technical replicates of n=1 with an average of 85 cells counted per sample.

Error bars represent mean with SEM.

Development • Supplementary information

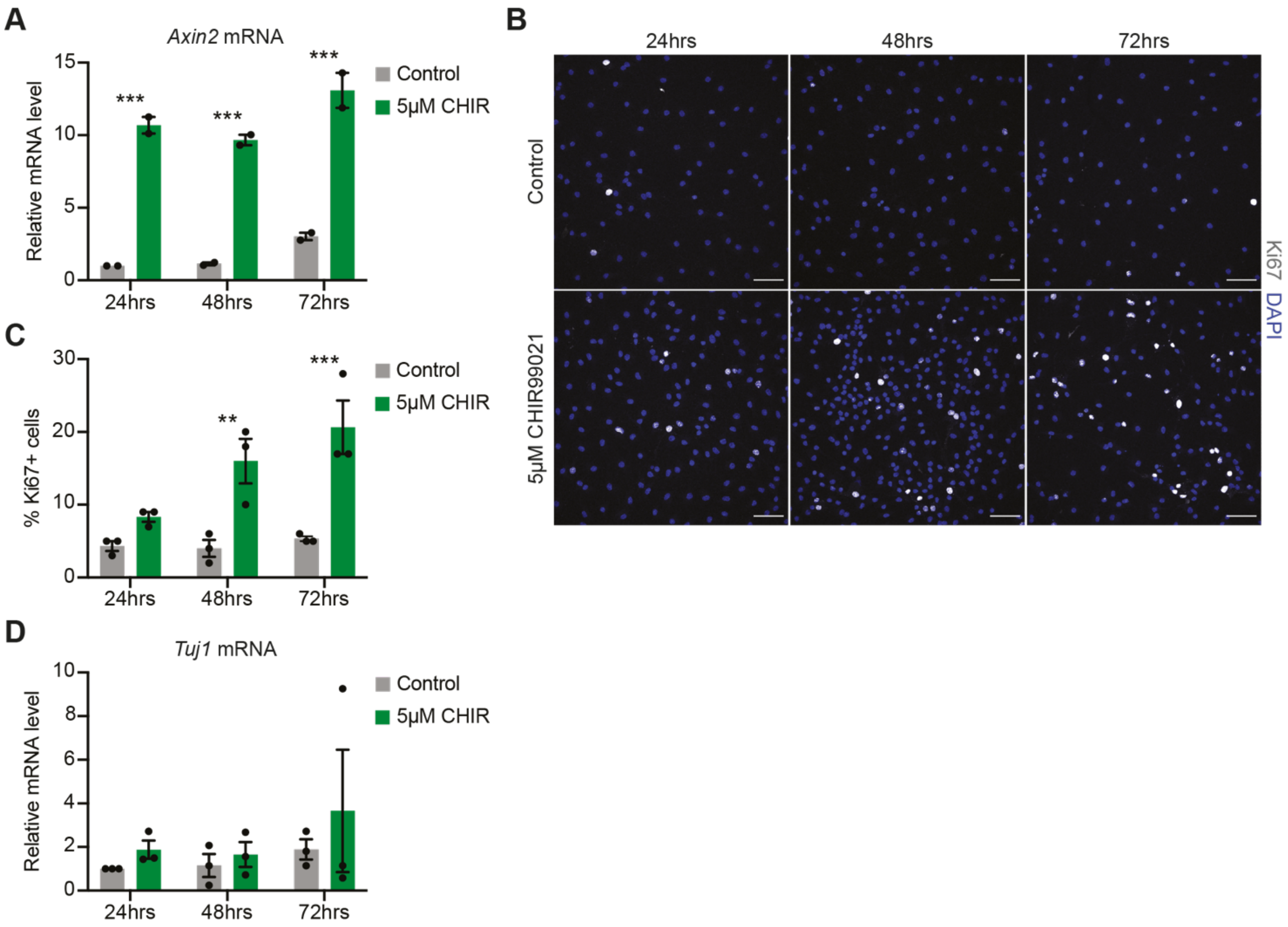

Fig. S7. Quiescent NSC activation is sustained by prolonged Wnt/β-catenin stimulation.

(**A**) Upregulation of *Axin2* is sustained by 5μM CHIR99021 treatment of quiescent NSCs for 24hrs, 48hrs and 72hrs. n=2.

(**B**) Immunolabelling of the proliferation marker Ki67 and DAPI in quiescent NSCs treated with 5μM CHIR99021 for 24hrs, 48hrs and 72hrs. Scale bars, 50μm.

(**C**) Quantification of the proportion of Ki67+ cells in (**B**). Proliferation increases with longer 5μM CHIR99021 treatments of quiescent NSCs (Control vs 5μM CHIR99021: 24hrs, 4.33 ± 0.67 vs 8.33 ± 0.67. 48hrs, 4 ± 1.16 vs 16 ± 3.06. 72hrs, 5.33 ± 0.33 vs 20.67 ± 3.67). n=3.

(**D**) *Tuj1* expression is not significantly upregulated by 5μM CHIR99021 treatments of quiescent NSCs over time. n=3.

Statistics: Two-way ANOVA with Sidak's multiple comparisons test (**A**, **C** and **D**). (ns, p<0.05. **, p<0.01. ***, p<0.001). Error bars represent mean with SEM.

Development • Supplementary information

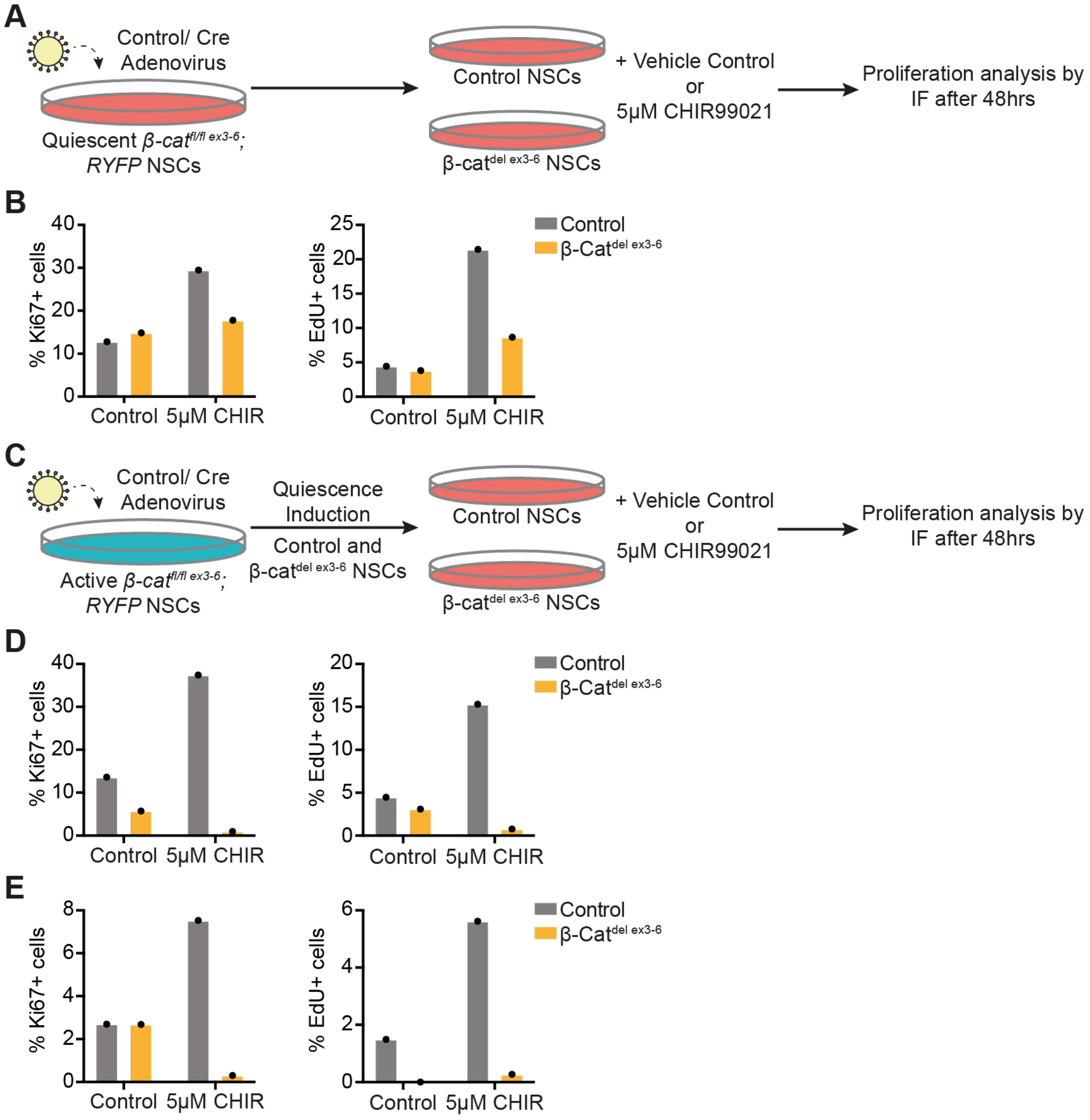

Fig. S8. Details of replicates comprising the data shown in Fig. 7E-I

(**A**) Quiescent β-cat$^{fl/fl\ ex3\text{-}6}$ NSCs were transduced with Control or Cre-adenovirus. Control and β-cat$^{del\ ex3\text{-}6}$ NSCs were then treated with either vehicle control or 5μM CHIR99021 for 48hrs before samples were collected for immunofluorescence analysis of proliferation markers.

(**B**) Quantification of the proportion of Ki67+ cells and the proportion of EdU+ cells in replicate 1 of the protocol detailed in (**A**).

(**C**) Quiescence was induced in control and β-cat$^{del\ ex3\text{-}6}$ NSCs 48hrs after transducing active β-cat$^{fl/fl\ ex3\text{-}6}$ NSCs with Control or Cre-adenovirus. After 72hrs of quiescence induction, Control and β-cat$^{del\ ex3\text{-}6}$ NSCs were treated with either vehicle control or 5μM CHIR99021 for 48hrs before samples were collected for immunofluorescence analysis of proliferation markers.

(**D, E**) Quantification of the proportion Ki67+ cells and the proportion of EdU+ cells in replicates 2 and 3 of the protocol detailed in (**C**).

See also the Materials & Methods section for further details.

Development • Supplementary information

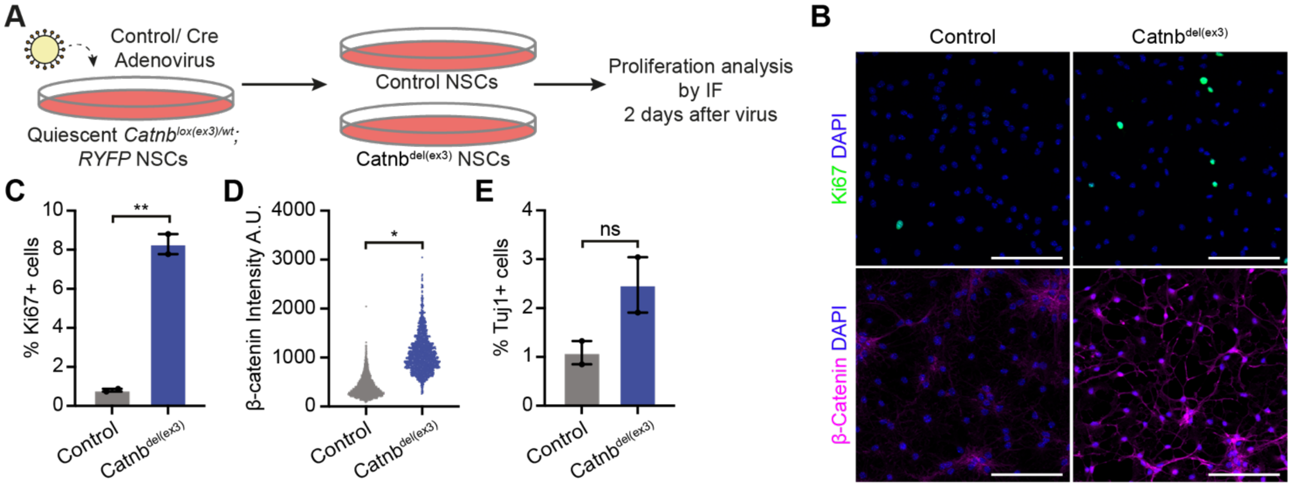

Fig. S9. Stabilisation of β-catenin in quiescent NSCs promotes their reactivation similarly to 5μM CHIR99021 treatment.

(**A**) Control and Catnb$^{del(ex3)}$ NSCs were taken 48hrs after transduction of quiescent Catnb$^{fl/wt(ex3)}$ NSCs with control and Cre adenovirus for immunofluorescence analysis.

(**B**) Ki67, β-catenin and DAPI immunolabelling in control and Catnb$^{del(ex3)}$ NSCs 48hrs after virus transduction. Scale bar, 100μm.

(**C**) Quantification of the data in (**B**). The proportion of Ki67+ cells is increased in Catnb$^{del(ex3)}$ NSCs (8.29 ± 0.51%) compared with control (0.81 ± 0.07%) indicating activation from quiescence upon stabilisation of β-catenin. n=2.

(**D**) Quantification of the data shown in (**B**) of the intensity of β-catenin staining in quiescent control and Catnb$^{del(ex3)}$ NSCs 48hrs after virus transduction. β-catenin intensity is increased in Catnb$^{del(ex3)}$ NSCs compared with control indicating stabilisation of β-catenin. A.U.= Arbitrary Units. n=2.

(**E**) The proportion of Tuj1+ cells is slightly increased in Catnb$^{del(ex3)}$ NSCs (2.48 ± 0.56%) compared with control (1.09 ± 0.24%) which is similar to the increase in Tuj1+ cells seen with 5μm CHIR99021 treatment in quiescent NSCs (Fig. 6F). n=2.

Statistics: unpaired two-tailed Student's t-test (**C, D and E**). (ns, p>0.05. *, p<0.05. **, p<0.01). Error bars represent mean with SEM.

Development • Supplementary information

Table S1. Primary and secondary antibodies.

Target Molecule	Species	Procedure	Dilution	Supplier	Catalogue #
Actin	Rabbit	WB	1:1000	Sigma-Aldrich	A2066
β-catenin	Mouse	IF-*in vivo*	1:100	BD Biosciences	610154
		IF-*in vitro*	1:250		
		WB	1:2000		
β-galactosidase	Chicken	IF	1:1000	Aves Lab Inc.	BGL-1010
CyclinD1	Rabbit	IF	1:25	ThermoScientific	RM-9104
DCX	Goat	IF	1:50	Santa-Cruz	Sc-8066 (Discontinued)
GFAP	Rat	IF	1:800	Invitrogen	13-0300
GFP	Chicken	IF	1:2000	Abcam	ab13970
Ki67	Mouse	IF	1:100	BD Biosciences	550609
Ki67	Rat	IF	1:200	Invitrogen	14-5698-82
Map2	Mouse	IF	1:200	Sigma	M4403
mCherry	Rabbit	IF	1:500	GeneTex	GTX128508
NeuN	Mouse	IF	1:800	Chemicon	MAB377
Prox1	Rabbit	IF	1:800	Merck	Ab5475
Sox2	Rat	IF	1:400	EBioscience	14-9811-82
Tbr2	Rabbit	IF	1:200	Abcam	ab183991
Tuj1	Rabbit	IF	1:400	Covance	PRB-435P
Tuj1	Mouse	IF	1:400	Covance	MMS-435P
Wnt7a	Rabbit	WB	1:500	Abcam	ab100792
Vimentin	Mouse	WB	1:2000	Sigma	V9131
Chicken IgG	Donkey	IF-488	1:500	Jackson	703-545-155
Mouse IgG	Donkey	IF-488	1:500	Jackson	715-546-151
Rat IgG	Donkey	IF-Cy3	1:500	Jackson	712-166-153
Rabbit IgG	Donkey	IF-Cy3	1:500	Jackson	711-166-152
Mouse IgG	Donkey	IF-Cy3	1:500	Jackson	715-166-151
Goat IgG	Donkey	IF-647	1:500	Jackson	705-605-147
Mouse IgG	Donkey	IF-647	1:500	Jackson	715-606-151
Rabbit IgG	Donkey	IF-647	1:500	Jackson	711-606-152
Rat IgG	Donkey	IF-647	1:500	Jackson	112-175-167
Mouse IgG	Rabbit	WB-HRP	1:1000	Dako	P0161
Rabbit IgG	Goat	WB-HRP	1:1000	Dako	P0448

Table S2. List of TaqMan probes from Applied Biosystems used for qPCR gene expression assays.

Gene	Assay ID	Catalogue Number
ActinB	Mm00607939_s1	4352933E
Ascl1	Mm03058063_m1	4331182
Axin2	Mm00443610_m1	4331182
GAPDH	Mm99999915_g1	4352932E
HopX	Mm00558630_m1	4331182
Id4	Mm00499701_m1	4331182
Nestin	Mm00450205_m1	4331182
Ngn2	Mm00437603_g1	4331182
Sox2	Mm00488369_s1	4331182
Tubb3 (Tuj1)	Mm00727586_s1	4331182
β-catenin exons 4-5	Mm01350386_g1	4351372
