# Supplementary material for: Wnt/β-catenin signalling is dispensable for adult neural stem cell homeostasis and activation
Source: Development. 2021 Oct 19;148(20):dev199629. doi: 10.1242/dev.199629 (PMC8572000; doi:10.1242/dev.199629)
Supplement: Reviewer comments [file develop-148-199629_review_history.pdf]

Wnt/ß-catenin signalling is dispensable for adult neural stem cell homeostasis and activation

Sophie H. L. Austin, Rut Gabarró-Solanas, Piero Rigo, Oana Paun, Lachlan Harris, Francois Guillemot and Noelia Urbán
DOI: 10.1242/dev.199629

Editor: Paola Arlotta

Review timeline

Original submission:	22 Mar 2021
Editorial decision:	29 Apr 2021
Second submission:	20 Aug 2021
Accepted:	17 Sep 2021

Reviewer 1

Evidence, reproducibility and clarity

Summary

Wnt/beta-catenin signaling has been studies in the context of adult neurogenesis for decades. It has been shown that modulation of Wnt signaling regulates adult neurogenesis, but the consequences were not always consistent. In this study, the authors developed conditional knockout mouse lines to test whether beta-catenin is essential for the regulation of adult neurogenesis.

First, using a published single cell seq-data and a reporter TG mouse system, they validated the expression of Wnt-pathway molecules in qNSCs and active NSCs. Then, beta-catenin conditional cKO mice were analyzed. The authors did not find any changes in total number of NSCs, the activation of NSCs, and the number of IPCs as well as neuroblasts.
Subsequently, using in vitro culture system, the authors addressed if the proliferation and differentiation are affected in vitro conditions. Both proliferation and activation from the quiescent state were not affected in cKO NSCs. Finally, they demonstrated that an artificial stimulation of Wnt signaling by CHIR can induce differentiation or proliferation depending on cellular states and doses, thus NSCs can respond to Wnt signaling. Based on these data, they concluded that beta-catenin is dispensable for the maintenance/activation of NSCs in vivo, although NSCs can respond to Wnt/beta-catenin signaling. Overall, the results are reliable and important for the field. However, several points need to be addressed and clarified to support their conclusion. I am hopeful that the authors find my comments helpful and constructive.

1. Validation of cKO in vivo.
Although the authors validated cKO of beta-catenin in vivo using FACS/qPCR at the transcript level, it would be important to check when and to what extent beta-catenin proteins are downregulated in qNSC/activeNSCs in vivo. This will be easily assessed by immunohistochemistry. In the same line, although the authors confirmed the reduction of beta-catenin signaling using beta-gal signaling in cKO mice, it would be important to check if this can be cross-checked by staining the nuclear localization of beta-catenin. This confirmation would strength the authors statement and clear that some remained beta- catenin at the plasma membrane may not be compensating their function.
Independent of the confirmation of beta-catenin cKO, it would be important to check if the downstream targets of Wnt/beta-catenin signals (ex. Expression of Axin2) were also attenuated. This point should be addressed both in vivo and in vitro.

© 2021. Published by The Company of Biologists under the terms of the Creative Commons Attribution License (https://creativecommons.org/licenses/by/4.0/).

2.Wnt/beta-catenin signals in qNSC and active NSC in vitro
The authors indicated that the depletion of beta-catenin had no effect on qNSCs and active NSCs in vitro. However, it is not clear whether Wnt/beta-catenin signaling is activated in their culture conditions. If there are no inputs of Wnt signaling in cultured cells, the depletion of beta-catenin will not lead any impacts. Therefore, it would be critical to check if the Wnt- signaling is activated in control cells in their culture condition, and if the downstream targets of Wnt-signaling are downregulated in cKO qNSCs/active NSCs.

3.ChIR treatment on cKO cells
The authors only use WT cells for ChIR treatment. To investigate whether the effect of ChIR come through the beta-catenin signaling pathway, why don't they use cKO NSCs for ChIR treatment (Fig5-7)?
4. Different Wnt signaling levels between in vivo and in vitro
The authors indicated that different levels of Wnt signaling could results in different outcomes based on in vitro observation. What are the levels of Wnt signaling in vivo compared to in vitro ChIR treatment? Activation of Wnt/beta-catenin in vivo is much weaker than in vitro CHIR treatment, therefore the contribution of Wnt signaling at endogenous levels is negligible? This may help to explain why Wnt/beta-catenin is dispensable in vivo, at least in young state. This can be addressed by probing the levels of downstream targets.

Significance

A genetic approach to address the role of Wnt/Beta-catenin signaling is critical for the field. The audience would be interested if this study make it clear previously reported discrepancy.

Reviewer 2

Evidence, reproducibility and clarity

Summary:

Wnt/beta-catenin signaling is considered central to the regulation of adult hippocampal neurogenesis. In this manuscript Austin and colleagues interrogate the function of beta-catenin-dependent signaling using in vivo beta-catenin conditional knockout and gain-of-function approaches combined with in vitro pharmacological and genetic approaches. The authors confirm previous reports of Wnt/beta-catenin signaling in adult hippocampal neurogenesis and report the surprising findings that

- Deletion of beta-catenin from stem cells does not affect stem cell numbers and their activation / proliferation in vivo and in vitro
- Deletion of beta-catenin from stem cells does not affect neuronal differentiation in vivo and in vitro

Moreover, the authors show that expression of a stabilized form of beta-catenin affects stem cell positioning in vivo and that the effects of treatment of cultured hippocampal stem/progenitor cells with a pharmacological stimulator of Wnt/beta-catenin signaling are dose and time-dependent. The authors discuss that their findings suggest that Wnt/beta-catenin signaling is dispensable for neural stem cell homeostasis and that Wnt/beta-catenin signaling may have a function in the response of stem cells to external stimuli.

Comments:
A major challenge is to separate cell adhesion functions of beta-catenin from its function in the canonical Wnt/beta-catenin signaling pathway. The authors tested two different conditional bcat alleles (bcatdel ex2-6 ; bcatdel ex3-6) to delete bcat from stem cells. It is a bit unfortunate that the authors chose to test two conditional alleles that would affect cell adhesion and transcriptional activity instead of the Ctnnb1dm allele (Draganova et al. 2015, Stem Cells), which would have been a cleaner way to specifically address the contribution of beta-catenin transcriptional activity in adult hippocampal neural stem cells. Was there a specific reason not to use the Ctnnb1dm conditional mice? Please comment / discuss.

© 2021. Published by The Company of Biologists under the terms of the Creative Commons Attribution License (https://creativecommons.org/licenses/by/4.0/).

The authors control for downregulation of beta-catenin signaling activity in the bcatdel ex2-6 through the analysis of the BATGAL reporter. 30 days after recombination, they observe a drop in reporter activity (from 31% to 13%). While this drop shows that at the time of analysis beta-catenin signaling activity was reduced, the lack of complete downregulation of reporter activity raises the issue whether long-term stability of the b-catenin protein may be a confounding factor at this time-point. In particular effects of b-catenin on the DCX population, which to a significant extent is generated several days to weeks before the time-point of analysis, may not be revealed. Data on the time-course of downregulation of the BATGAL reporter could help for the interpretation of the data as would analysis of beta-catenin protein levels in recombined cells. In addition, analysis of bcatdel ex2-6 at a later time-point after recombination, at which beta-catenin signaling activity is further downregulated, would strengthen the surprising finding that loss of beta-catenin signaling activity does not hamper neuronal differentiation in the adult hippocampus.

Was quantification performed only in recombined (i.e., reporter positive) cells or in recombined and non-recombined cells? I could not locate that information. Given the evidence for feed-back regulation from intermediate precursor cells / immature neurons to stem cells (e.g. Lavado et al. 2010, Plos Biology), it is important to separately evaluate the development of recombined and non-recombined cells to evaluate the behavior of beta- catenin signaling deficient stem cells.

Reports from (Kuwabara et al. 2009, Nat Neurosci), (Gao et al. 2009, Nat Neurosci) and (Karalay et al. 2011, PNAS) suggest that beta-catenin signaling activity drives dentate granule neuron identity through regulating the expression of Neurod1 and Prox1. Given that in these studies neither loss of Neurod1 nor of Prox1 affects neuronal fate commitment but long-term survival and that the studies by (Gao et al. 2007, J Neurosci) and (Heppt et al. 2020, EMBO J) suggest that loss-of-beta-catenin affects neuronal survival, it may be interesting to evaluate a) whether a dentate granule neuron identity, b) long-term survival of adult generated neurons are affected. At the minimum these studies should be more extensively discussed.

It has been suggested that the neural stem cell population in the adult hippocampus may be heterogenous with one population being responsible for baseline neurogenesis and being resistant to age-associated depletion and a second population driving high levels of neurogenesis in young adults (see also Urban, Bloomfield and Guillemot 2019, Neuron). The observation that beta-catenin signaling is only active in a small fraction of stem cells and their progeny raises the question whether it fulfills only a function in a specific subpopulation. Such possibility should at least be discussed.

The recently published studies by (Rosenbloom et al. 2020, PNAS) and (Heppt et al. 2020, EMBO J) strongly suggest that beta-catenin signaling dynamics are critical for the regulation / modulation of adult hippocampal neurogenesis. The aspect of beta-catenin signaling dynamics should be discussed.

Significance

Adult neurogenesis is considered an important factor in hippocampal plasticity and its disturbance is thought to contribute to the pathogenesis in several psychiatric and degenerative diseases. Wnt/beta-catenin signaling is considered central to the regulation of adult hippocampal neurogenesis. In this regard, the manuscript describes the potentially very important and surprising finding that deletion of beta-catenin from neural stem cells does not generate major neurogenesis phenotypes. The concern with the present manuscript is, that the lack of phenotype requires additional analyses to exclude that phenotypes develop with a delay because of long-term stability of the beta-catenin protein.

The significance of the manuscript and its interest to a wider audience would in addition be greatly enhanced, if the authors could provide some mechanistic data that would explain the discrepancies between published functions of Wnt/beta-catenin-signaling dependent regulation of neurogenesis and their own findings. The manuscript would also gain significance if the authors would provide solid data for their interesting hypothesis that beta- catenin-signaling contributes to the regulation of adult hippocampal neurogenesis in response to extrinsic stimuli. In this regard one potential approach would be to analyse whether extrinsic stimuli such as running would be able stimulate the activation of stem cells.

© 2021. Published by The Company of Biologists under the terms of the Creative Commons Attribution License (https://creativecommons.org/licenses/by/4.0/).

Expertise:

Adult neurogenesis, stem cell biology, signaling

Author response to reviewers' comments

Response to Reviewers

Response to reviewers updated after revision

In order to make the revisions easy to follow, we have added our new results and changes after the text of our initial response to reviewers. New text appears in purple.

Summary:

We want to thank the reviewers for their careful evaluation of our work and their helpful suggestions. We provide at the end of this letter a point by point response of how we aim to address their concerns, which can be summarised in the following main points:

1. We will provide further evidence for the efficiency and dynamics of beta-catenin deletion in adult neural stem cells *in vivo* (point raised by both reviewers).
We fully agree that although we tested for the disappearance of beta-catenin transcripts in sorted NSCs after deletion, providing further proof of the absence of beta-catenin protein in these cells will help strengthen our conclusions. For this, we are performing additional stainings for beta-catenin and Wnt/beta-catenin targets, together with neural stem cell markers, to quantify the loss of beta-catenin and Wnt/beta-catenin signalling in NSCs at P90 (30 days after deletion), as well as new P150 samples (90 days after deletion).
We have been able to show that beta-catenin protein is absent in recombined NSCs by 30 days after deletion (Fig. S2F). However, we could not see a reduction in Wnt-target genes (Axin2 and CyclinD1) upon beta-catenin deletion. We also show that beta-catenin is expressed at very low levels in the DG of young mice (Fig. S2D), which could explain the lack of effect of its deletion both on downstream target genes and on NSC behaviour at this stage.

2. We will investigate in further detail the effects (or lack of effect) of beta-catenin deletion on adult neurogenesis.
The focus of our work is the effect of Wnt/beta-catenin signalling on NSCs. Nevertheless, we agree with reviewer 2 that extending our analysis to later stages in the neurogenic process will be of importance to better contrast our results with previous reports identifying a role for Wnt in neuronal production in the adult hippocampus. We are currently processing new material from mice in which beta-catenin was deleted at P60 and brains collected after 3 months to evaluate the long-term effects of beta-catenin deletion on the neurogenic output of NSCs. We will also perform stainings of Wnt-responsive neuronal genes, such as NeuroD1 and Prox1, at P90 and P150 in both control and beta-catenin cKO mice.
Our new results evaluating beta-catenin loss after 3 months of recombination confirm that NSC maintenance and neurogenesis are not affected in beta-catenin cKO mice (Fig. S3).

3. We are aiming to confirm that the *in vitro* effects of CHIR99021 on NSCs are mediated by beta-catenin. We already provide evidence that stimulation with Wnt3a has the same effect as inhibition of GSK3beta by CHIR99021. To further prove the link of the observed effects to Wnt-beta-catenin signalling, we will repeat some of our key experiments using beta-catenin floxed cells (both induction of neuronal differentiation and re-activation from quiescence) as reviewer 1 suggests.
We have now confirmed that re-activation of quiescent cells upon CHIR stimulation requires beta-catenin, since the effect is totally abrogated in beta-catenin cKO cells (new data in Figure 7 and new Supplementary Figure S8):

© 2021. Published by The Company of Biologists under the terms of the Creative Commons Attribution License (https://creativecommons.org/licenses/by/4.0/).

Summary of revisions: Taken together, our new results have strengthened our conclusion that Wnt/beta-catenin signalling is not necessary for NSC homeostasis and neurogenesis in the adult DG in young animals. We also further demonstrate that quiescent NSCs can be reactivated upon Wnt stimulation in a beta-catenin dependent manner.
We want to thank again the reviewers for helping us improve our manuscript and hope they will find it now suitable for publication.

List of changes:
New and updated text appears in purple also in the re-submitted manuscript.
Summary of changes to the figures:
Old Figure S1 is now split into Figures S1 and S2, with S2 including new data on the comparison of beta-catenin levels between SVZ and DG, the levels of *Axin2* upon beta-catenin deletion and the immunohistochemistry showing efficient deletion of beta-catenin in DG NSCs.
A new Figure S3 has been added with data on the long-term deletion of beta-catenin *in vivo*. In Figure 4, a new panel, L, has been added to show the lack of effect of beta-catenin deletion on the induction of quiescence in NSCs in vitro.

Former Figure 7 is now Supplementary Figure 7.
Data in former Figure S5 (effects of rWnt3 on re-activation of quiescent NSCs) is now shown in new Figure 7, which also includes the new data on the lack of re-activation of quiescent NSCs upon beta-catenin deletion. New Supplementary Figure S8 contains details of the individual experiments, which are shown combined in Figure 7.
Finally, data on the re-activation of quiescent NSCs upon beta-catenin stabilisation is shown in new Supplementary Figure S9.
Figure numbers and in-text references to the figures, as well as figure legends, have been updated throughout to match the new layout.

Reviewer #1 (Evidence, reproducibility and clarity (Required)):

Summary

Wnt/beta-catenin signaling has been studies in the context of adult neurogenesis for decades. It has been shown that modulation of Wnt signaling regulates adult neurogenesis, but the consequences were not always consistent. In this study, the authors developed conditional knockout mouse lines to test whether beta-catenin is essential for the regulation of adult neurogenesis.

First, using a published single cell seq-data and a reporter TG mouse system, they validated the expression of Wnt-pathway molecules in qNSCs and active NSCs. Then, beta-catenin conditional cKO mice were analyzed. The authors did not find any changes in total number of NSCs, the activation of NSCs, and the number of IPCs as well as neuroblasts. Subsequently, using in vitro culture system, the authors addressed if the proliferation and differentiation are affected in vitro conditions. Both proliferation and activation from the quiescent state were not affected in cKO NSCs. Finally, they demonstrated that an artificial stimulation of Wnt signaling by CHIR can induce differentiation or proliferation depending on cellular states and doses, thus NSCs can respond to Wnt signaling. Based on these data, they concluded that beta- catenin is dispensable for the maintenance/activation of NSCs in vivo, although NSCs can respond to Wnt/beta-catenin signaling. Overall, the results are reliable and important for the field. However, several points need to be addressed and clarified to support their conclusion. I am hopeful that the authors find my comments helpful and constructive.

Many thanks for your insightful comments, we believe they will indeed help us improve our manuscript.

1. Validation of cKO in vivo.
Although the authors validated cKO of beta-catenin in vivo using FACS/qPCR at the transcript level, it would be important to check when and to what extent beta-catenin proteins are downregulated in qNSC/activeNSCs in vivo. This will be easily assessed by immunohistochemistry. In the same line, although the authors confirmed the reduction of beta- catenin signaling using beta-gal signaling in cKO mice, it would be important to check if this can be cross-checked by staining the

© 2021. Published by The Company of Biologists under the terms of the Creative Commons Attribution License (https://creativecommons.org/licenses/by/4.0/).

nuclear localization of beta-catenin. This confirmation would strength the authors statement and clear that some remained beta-catenin at the plasma membrane may not be compensating their function. Independent of the confirmation of beta-catenin cKO, it would be important to check if the downstream targets of Wnt/beta-catenin signals (ex. Expression of Axin2) were also attenuated. This point should be addressed both in vivo and in vitro.

We are performing immunohistochemistry and quantification of beta-catenin in control and cKO brain samples, as suggested by the reviewer. Unfortunately, we have not yet found an antibody and labelling protocol that allows us to detect nuclear beta-catenin, even in control samples, so with our current antibody, we won't be able to show a reduction in nuclear localization of beta-catenin in the cKO samples. We are testing alternative beta-catenin antibodies that could help us overcome this limitation. As the reviewer mentions, we do see a reduction in reporter expression in BATGAL mice upon deletion of beta-catenin. In order to further demonstrate effective Wnt signalling attenuation in our mutant mice we are testing antibodies for Wnt targets such as Axin2, CcnD1 and NeuroD1.

We tested three beta-catenin antibodies (BD BioSciences, 610154. Sigma, C2206. Millipore/Upstate 05-665) but unfortunately none of these allowed the detection of beta-catenin in the nucleus of NSCs at basal levels. Even the antibody that gave the best signal to noise ratio (BD BioSciences, 610154) failed to provide reliable staining in the DG in our brain samples. In contrast, beta-catenin staining was consistently strong and robust in the other main neurogenic area, the SVZ. We now show this difference to illustrate the very low basal levels of beta-catenin present in the DG (shown here and in Fig. S2D).

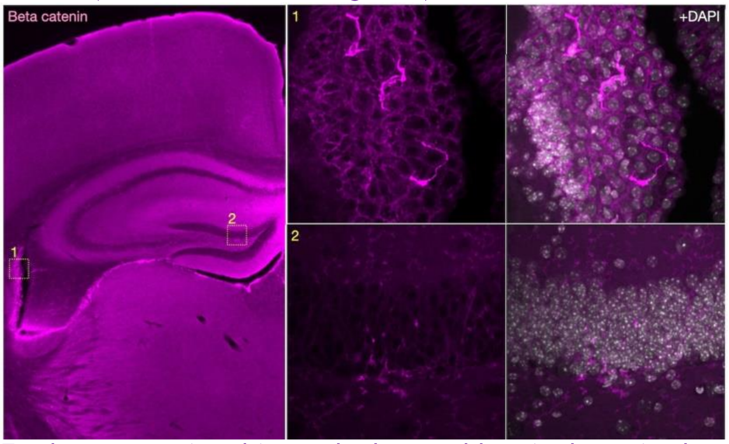

Further supporting this, we had no problem in detecting beta-catenin protein in the DG of the Catnb$^{del(ex3)}$ mice, in which beta-catenin is stabilised (Fig. S4B). Despite the unreliability of the staining, we did manage to show that beta-catenin protein is not present in recombined NSCs of Βcat$^{del\ ex2\text{-}6}$ mice. To show this clearly, we chose a region of the DG in which an unrecombined NSC (YFP-negative, beta-catenin-positive) is seen alongside recombined NSCs (YFP-positive, beta-catenin-negative). These data, shown below, have now been included in the manuscript as Supplementary Figure 2E and F.

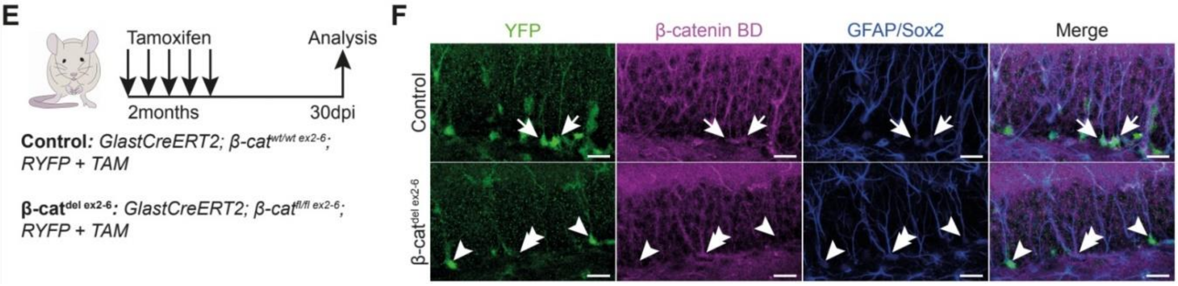

We also tested antibodies for Axin2, CyclinD1 and NeuroD1, of which only CyclinD1 worked in our hands. Quantification of CyclinD1 in control and Βcat$^{del\ ex2\text{-}6}$ samples showed no difference between the genotypes (results below, not included in the manuscript).

© 2021. Published by The Company of Biologists under the terms of the Creative Commons Attribution License (https://creativecommons.org/licenses/by/4.0/).

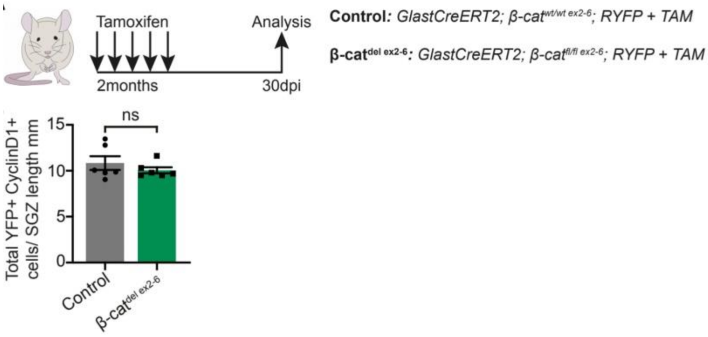

We also performed qPCR for *Axin2* in FACS-sorted NSCs, where we showed a clear reduction of beta-catenin transcripts in the beta-catenin cKO cells. Again, no difference between genotypes was observed (results below and now included in Figure S2G).

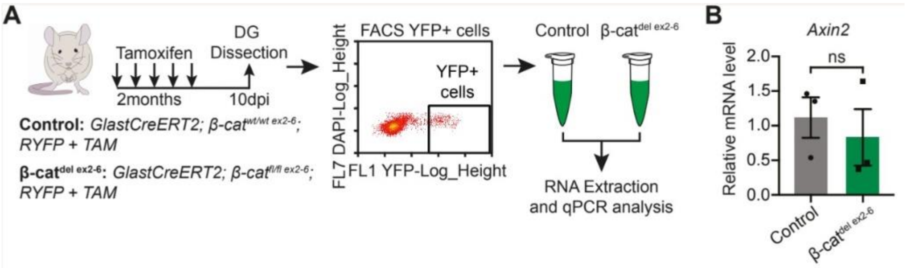

While surprising, we observed the same lack of effect of beta-catenin deletion in NSCs *in vitro*, including on *Axin2* levels. In Figure S5, we demonstrate that beta-catenin protein is efficiently deleted by 48 hours and that this fully blocks the response of NSCs to CHIR stimulation. However, basal levels of *Axin2* remained unchanged in these cells, as they do *in vivo* (Figure S5C).
These results together have led us to make a much stronger case of the lack of significant activation of the Wnt/beta-catenin pathway in DG NSCs in young animals (Results section: page 6 and page 9; Discussion section: page 12) and have strengthened our conclusion that beta-catenin signalling is dispensable for NSC homeostasis.

2. Wnt/beta-catenin signals in qNSC and active NSC in vitro.
The authors indicated that the depletion of beta-catenin had no effect on qNSCs and active NSCs in vitro. However, it is not clear whether Wnt/beta-catenin signaling is activated in their culture conditions. If there are no inputs of Wnt signaling in cultured cells, the depletion of beta-catenin will not lead any impacts. Therefore, it would be critical to check if the Wnt- signaling is activated in control cells in their culture condition, and if the downstream targets of Wnt-signaling are downregulated in cKO qNSCs/active NSCs.

We agree that this is an important conceptual point that needs to be clarified. From our data (see Figure S3C), we can see that deletion of beta-catenin in NSCs *in vitro* blocks their response to Wnt stimulation (with CHIR99021) but it did not lower the levels of *Axin2*. From this, we can deduce that Wnt signalling is indeed not significantly activated in proliferating NSCs *in vitro*, despite the expression of Wnt ligands by these cells (Figure 3). We will perform further analysis of Wnt target genes in control and cKO NSCs *in vitro* to confirm this observation. Of note, the lack of Wnt signalling activity in NSCs would further support our claim that Wnt is dispensable for their proliferation and maintenance. We will make this point clearer in the manuscript.
This point is tightly related to the previous one. In addition, we performed QPCR for *Axin2* (A) and *CyclinD1* (B) in recombined beta-catenin cKO cells *in vitro* and saw no difference in their levels respect to control cells (Figure shown here, not included in the manuscript). This confirms our hypothesis that Wnt signalling is barely active in these cells, a point we have made clearer in the text (Results section: page 6 and page 9; Discussion section: page 12).

© 2021. Published by The Company of Biologists under the terms of the Creative Commons Attribution License (https://creativecommons.org/licenses/by/4.0/).

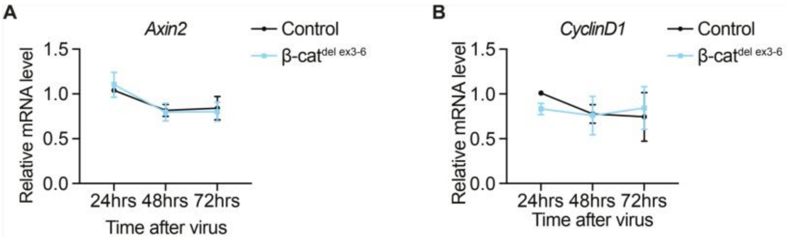

3. ChIR treatment on cKO cells.

The authors only use WT cells for ChIR treatment. To investigate whether the effect of ChIR come through the beta-catenin signaling pathway, why don't they use cKO NSCs for ChIR treatment (Fig5-7)?

This is a great suggestion and we are performing these experiments with control and cKO NSCs. We have repeated the key experiment, where quiescent NSCs re-activate upon treatment with 5uM CHIR, using cKO NSCs. Our results clearly show that beta-catenin mediates the response of NSCs to CHIR, as cKO NSCs fail to re-activate. We also generated a NSC line from the DG of Catnb$^{lox(ex3)}$ mice, induced quiescence in these cells and then stabilised beta-catenin by adding CRE. Our results demonstrate that beta-catenin stabilisation is sufficient to overcome BMP-induced quiescence in NSCs. These data are depicted below and now shown in Figures 7, S8 and S9.

Beta-catenin cKO NSCs (Figures 7 and S8):

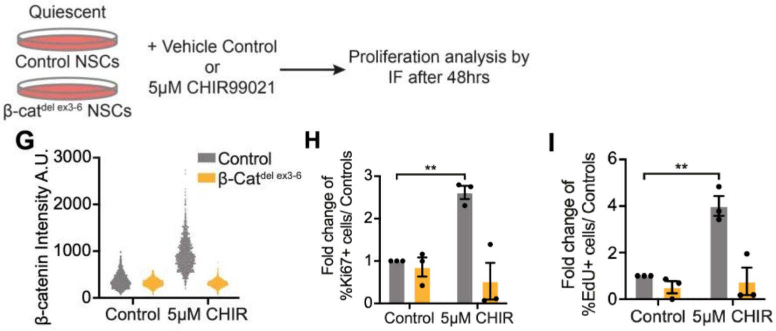

Catnb$^{lox(ex3)}$ NSCs (Figure S9):

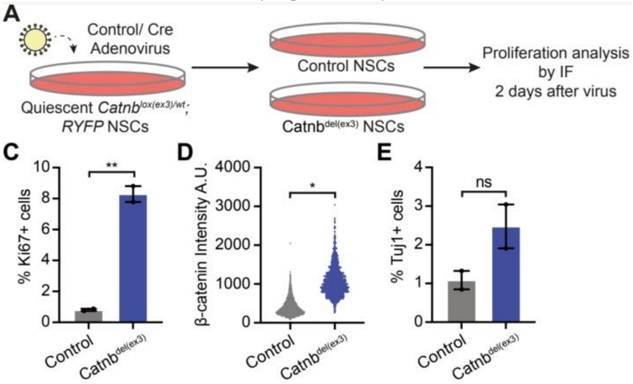

4. Different Wnt signaling levels between in vivo and in vitro.

The authors indicated that different levels of Wnt signaling could results in different outcomes based on in vitro observation. What are the levels of Wnt signaling in vivo compared to in vitro ChIR treatment? Activation of Wnt/beta-catenin in vivo is much weaker than in vitro CHIR treatment, therefore the contribution of Wnt signaling at endogenous levels is negligible? This may help to explain why Wnt/beta-catenin is dispensable in vivo, at least in young state. This can be addressed by probing the levels of downstream targets.

Levels of Wnt signalling are indeed central to our conclusions and we agree that a comparison of Wnt/beta-catenin signalling levels between our *in vitro* interventions and the *in vivo* situation would be important. However, we find that directly comparing the levels of downstream Wnt targets

© 2021. Published by The Company of Biologists under the terms of the Creative Commons Attribution License (https://creativecommons.org/licenses/by/4.0/).

between the two systems might prove challenging due to differences in methodology (immunolabeling is not a reliably quantitative method, especially when performed on such different sample types, with different fixation conditions, etc). We will nevertheless attempt such quantifications using immunolabelings for CcnD1, Axin2 and NeuroD1 both *in vivo* and *in vitro*. We also want to point out that CHIR is not the only way in which we have stimulated Wnt signalling in NSCs *in vitro*. In Figure S5, we demonstrate that treatment with Wnt3a can reactivate quiescent neural stem cell in a dose-dependent manner, showing that the effect of Wnt signalling on NSCs can be achieved also with a more physiological intervention.
Unfortunately, we were not able to find an appropriate way to compare the levels of Wnt activation between NSCs *in vivo* and *in vitro*. We nevertheless hope we have provided enough evidence that endogenous Wnt/beta-catenin signalling levels are extremely low in NSCs both *in vivo* and *in vitro*.

Reviewer #1 (Significance (Required)):

Significant.

A genetic approach to address the role of Wnt/Beta-catenin signaling is critical for the field. The audience would be interested if this study make it clear previously reported discrepancy.

Reviewer #2 (Evidence, reproducibility and clarity (Required)):

Summary:

Wnt/beta-catenin signaling is considered central to the regulation of adult hippocampal neurogenesis. In this manuscript Austin and colleagues interrogate the function of beta-catenin-dependent signaling using in vivo beta-catenin conditional knockout and gain-of-function approaches combined with in vitro pharmacological and genetic approaches. The authors confirm previous reports of Wnt/beta-catenin signaling in adult hippocampal neurogenesis and report the surprising findings that

- Deletion of beta-catenin from stem cells does not affect stem cell numbers and their activation / proliferation in vivo and in vitro
- Deletion of beta-catenin from stem cells does not affect neuronal differentiation in vivo and in vitro

Moreover, the authors show that expression of a stabilized form of beta-catenin affects stem cell positioning in vivo and that the effects of treatment of cultured hippocampal stem/progenitor cells with a pharmacological stimulator of Wnt/beta-catenin signaling are dose and time-dependent. The authors discuss that their findings suggest that Wnt/beta-catenin signaling is dispensable for neural stem cell homeostasis and that Wnt/beta-catenin signaling may have a function in the response of stem cells to external stimuli.

Comments:

A major challenge is to separate cell adhesion functions of beta-catenin from its function in the canonical Wnt/beta-catenin signaling pathway. The authors tested two different conditional bcat alleles (bcatdel ex2-6 ; bcatdel ex3-6) to delete bcat from stem cells. It is a bit unfortunate that the authors chose to test two conditional alleles that would affect cell adhesion and transcriptional activity instead of the Ctnnb1dm allele (Draganova et al. 2015, Stem Cells), which would have been a cleaner way to specifically address the contribution of beta-catenin transcriptional activity in adult hippocampal neural stem cells. Was there a specific reason not to use the Ctnnb1dm conditional mice? Please comment / discuss.

We agree with the reviewer that the Ctnnb1dm allele would better differentiate between cell adhesion and transcriptional effects of beta-catenin deletion. However, as we see no effect of beta-catenin deletion, we did not find it necessary to further dissect the differential contribution of cell adhesion and the Wnt/beta-catenin pathway in this particular case. We will add a comment on this point to the discussion.
We have added a comment on this point in page 14 of the discussion section:
"Interestingly, deletion of beta-catenin using the beta-catdel ex2-6 mice (Brault et al., 2001) did

© 2021. Published by The Company of Biologists under the terms of the Creative Commons Attribution License (https://creativecommons.org/licenses/by/4.0/).

not appear to affect cell-cell contacts and NSC positioning despite the potential of this mouse model to disrupt the function of beta-catenin in cell adhesion in addition to transcriptional regulation. This could be due to the differences between inhibition of baseline Wnt/beta-catenin signalling versus gain-of-function effects or to a compensation from beta- catenin, which has been shown to be able to replace beta-catenin's function at adherens junctions in hepatocytes (Wickline et al., 2013). Nevertheless, the lack of phenotype suggests that neither of beta-catenin functions in cell adhesion or transcriptional regulation are crucial for NSC homeostasis in the DG and precluded using other available conditional mice that would address the specific contribution of distinct beta-catenin domains to its function (Draganova et al., 2015)."

The authors control for downregulation of beta-catenin signaling activity in the bcatdel ex2-6 through the analysis of the BATGAL reporter. 30 days after recombination, they observe a drop in reporter activity (from 31% to 13%). While this drop shows that at the time of analysis beta-catenin signaling activity was reduced, the lack of complete downregulation of reporter activity raises the issue whether long-term stability of the b-catenin protein may be a confounding factor at this time-point. In particular effects of b-catenin on the DCX population, which to a significant extent is generated several days to weeks before the time-point of analysis, may not be revealed. Data on the time-course of downregulation of the BATGAL reporter could help for the interpretation of the data as would analysis of beta-catenin protein levels in recombined cells. In addition, analysis of bcatdel ex2-6 at a later time-point after recombination, at which beta-catenin signaling activity is further downregulated, would strengthen the surprising finding that loss of beta-catenin signaling activity does not hamper neuronal differentiation in the adult hippocampus.

We will monitor the disappearance of beta-catenin using immunohistochemistry for beta- catenin and downstream targets of Wnt in control and cKO brains, both at P90 and at a longer time after deletion (P150), as the reviewer suggests. Of note, when we deleted beta-catenin *in vitro* in NSCs, we could confirm the disappearance of the protein by 48 hours, and therefore beta-catenin stability cannot explain the lack of effect of the deletion (Figure S3B).
As stated in our response to point 1 of reviewer#1, despite the unreliability of the beta-catenin antibodies in detecting signal in the DG, we did manage to show that beta-catenin protein is not present in recombined NSCs in the $\beta cat^{del\ ex2\text{-}6}$ mice 30 days after tamoxifen administration. To show this clearly, we chose a region of the DG in which an unrecombined NSC (YFP- negative, beta-catenin-positive) is seen alongside recombined NSCs (YFP-positive, beta- catenin-negative) in the DG of $\beta cat^{del\ ex2\text{-}6}$ mice. These data are shown above in our response to reviewer #1 and have now been included in the manuscript as Supplementary Figure S2F.

Was quantification performed only in recombined (i.e., reporter positive) cells or in recombined and non-recombined cells? I could not locate that information. Given the evidence for feed-back regulation from intermediate precursor cells / immature neurons to stem cells (e.g. Lavado et al. 2010, Plos Biology), it is important to separately evaluate the development of recombined and non-recombined cells to evaluate the behavior of beta-catenin signaling deficient stem cells.

The quantifications were always performed in YFP+ recombined cells. The efficiency of recombination was very high (from 83 to 97%), therefore allowing no room for confounding effects of unrecombined cells. We will convey this information in a clearer way in our revised manuscript.
We have added mentions to the recombination of the YFP cassette in page 6 and the M&M (page 23), where we also further explain that our quantifications were always performed in YFP-positive cells.

Reports from (Kuwabara et al. 2009, Nat Neurosci), (Gao et al. 2009, Nat Neurosci) and (Karalay et al. 2011, PNAS) suggest that beta-catenin signaling activity drives dentate granule neuron identity through regulating the expression of Neurod1 and Prox1. Given that in these studies neither loss of Neurod1 nor of Prox1 affects neuronal fate commitment but long-term survival and that the studies by (Gao et al. 2007, J Neurosci) and (Heppt et al. 2020, EMBO J) suggest that loss-of-beta-catenin affects neuronal survival, it may be interesting to evaluate a) whether a dentate granule neuron identity, b) long-term survival of adult generated neurons are affected. At the minimum these studies should be more extensively discussed.

© 2021. Published by The Company of Biologists under the terms of the Creative Commons Attribution License (https://creativecommons.org/licenses/by/4.0/).

As mentioned in our response summary, our main aim is to test the effects of Wnt/beta-catenin signalling on NSCs. Nevertheless, these are excellent suggestions and we are currently performing immunohistochemistry for NeuroD1 and Prox1 to test whether they are downregulated in cKO brain samples. We have also performed a longer deletion of beta-catenin (deletion at P60 and analysis at P150) to test whether neurogenesis is affected in the cKO mice in the longer term.
Our results from the analysis at P150 of deletion of beta-catenin show no effect on neurogenesis by this late point either. Similarly, Prox1 levels are equal between Control and cKO DG at this time point. NeuroD1 staining unfortunately did not work in our hands. These results are now shown below and in new Figure S3.

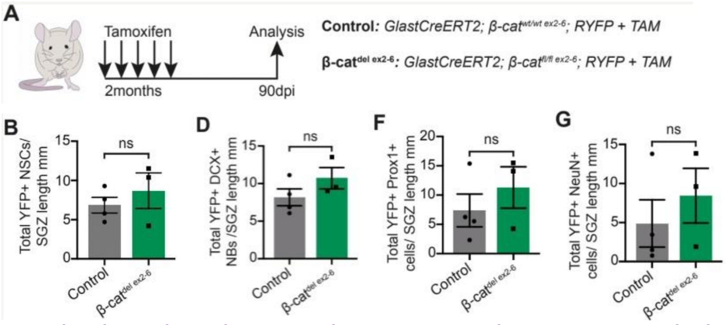

We also broadened up our discussion on this topic to include the additional references mentioned by the reviewer (page 13):
"This contradicts previous work from several labs showing that neuronal differentiation is impaired following the loss of beta-catenin signalling in proliferative progenitors or post- mitotic neuroblasts (Kuwabara et al., 2009, Karalay et al., 2011; Gao et al., 2007, Heppt et al., 2020). A possible explanation could be that Glast-dependent deletion occurs at a much earlier time point (when NSCs are still quiescent) compared with the acute loss of Wnt/beta-catenin signalling performed in previous studies, therefore allowing more time for compensatory mechanisms. Of note, while we did not observe a difference in neurogenesis, we cannot exclude that beta-catenin is needed for the functional maturation or integration of newly generated neurons.

It has been suggested that the neural stem cell population in the adult hippocampus may be heterogenous with one population being responsible for baseline neurogenesis and being resistant to age-associated depletion and a second population driving high levels of neurogenesis in young adults (see also Urban, Bloomfield and Guillemot 2019, Neuron). The observation that beta-catenin signaling is only active in a small fraction of stem cells and their progeny raises the question whether it fulfills only a function in a specific subpopulation. Such possibility should at least be discussed."

This is a very interesting point, which we will include in the discussion of our revised manuscript. We are also performing immunohistochemistry for Id4 together with beta-catenin or downstream targets of Wnt and NSC markers to determine whether the resting population (described in Urban et al. 2016 and Harris et al. 2021), which has low levels of Id4 is more responsive to Wnt than the dormant population.
We now show that there is no correlation between Id4 (low in resting NSCs) and beta-galactosidase levels in Id4-positive NSCs in BATGAL mice (Figure 1F, G).

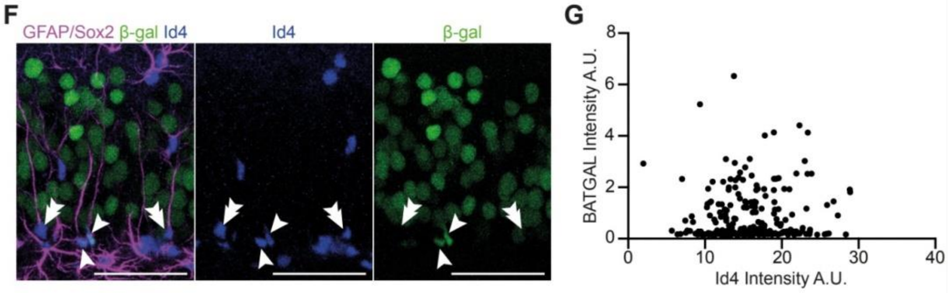

We also included a small paragraph in the discussion referring to this point (page 15):
"Only a small fraction of NSCs show active Wnt signaling at any time point. We initially hypothesised this might be linked to their activation status, but our results show that Wnt signaling is not correlated to NSC activation. The cause behind the heterogeneous response of

© 2021. Published by The Company of Biologists under the terms of the Creative Commons Attribution License (https://creativecommons.org/licenses/by/4.0/).

NSCs to Wnt remains to be determined, with possible contributors being oscillations in Wnt signalling, varying local levels of Wnt or the existence of a specific Wnt-responding NSC population."

The recently published studies by (Rosenbloom et al. 2020, PNAS) and (Heppt et al. 2020, EMBO J) strongly suggest that beta-catenin signaling dynamics are critical for the regulation / modulation of adult hippocampal neurogenesis. The aspect of beta-catenin signaling dynamics should be discussed.

We will include a discussion of beta-catenin signalling dynamics in the revised version of the manuscript.
This point can be found in the discussion section, page 16:
"In addition, two recent papers have highlighted the importance of beta-catenin signalling dynamics in determining Wnt function during adult neurogenesis (Heppt et al., 2020; Rosenbloom et al., 2020). Therefore, Wnt/beta-catenin signalling levels and/or dynamics modulated by external stimuli could play a key role in determining the response of quiescent NSCs to other niche cues."

Reviewer #2 (Significance (Required)):

Significance:

Adult neurogenesis is considered an important factor in hippocampal plasticity and its disturbance is thought to contribute to the pathogenesis in several psychiatric and degenerative diseases. Wnt/beta-catenin signaling is considered central to the regulation of adult hippocampal neurogenesis. In this regard, the manuscript describes the potentially very important and surprising finding that deletion of beta-catenin from neural stem cells does not generate major neurogenesis phenotypes. The concern with the present manuscript is, that the lack of phenotype requires additional analyses to exclude that phenotypes develop with a delay because of long-term stability of the beta-catenin protein.

We believe the revisions outlined above will address these concerns.

The significance of the manuscript and its interest to a wider audience would in addition be greatly enhanced, if the authors could provide some mechanistic data that would explain the discrepancies between published functions of Wnt/beta-catenin-signaling dependent regulation of neurogenesis and their own findings. The manuscript would also gain significance if the authors would provide solid data for their interesting hypothesis that beta-catenin-signaling contributes to the regulation of adult hippocampal neurogenesis in response to extrinsic stimuli. In this regard one potential approach would be to analyse whether extrinsic stimuli such as running would be able stimulate the activation of stem cells.

Both finding a mechanism to explain the observed discrepancies and demonstrating that Wnt has a role in the response of NSCs to extrinsic stimuli are excellent follow-up suggestions to our work and we thank the reviewer for these recommendations. However, addressing these points would take many months (if not years) and is not necessary to support the current conclusions of our work. We therefore believe they are out of the scope of this current manuscript.

Expertise:

Adult neurogenesis, stem cell biology, signaling

Original submission

First decision letter

MS ID#: DEVELOP/2021/199629

© 2021. Published by The Company of Biologists under the terms of the Creative Commons Attribution License (https://creativecommons.org/licenses/by/4.0/).

MS TITLE: Wnt/beta-catenin signalling is dispensable for adult neural stem cell homeostasis and activation
AUTHORS: Sophie H L Austin, Lachlan Harris, Oana Paun, Piero Rigo, Francois Guillemot, and Noelia Urban Avellaneda

Thank you for transferring your paper to Development from Review Commons. My deepest apologies for the delay in returning to you. I have had a look at your paper, at the reviews and at your revision plan, and the revisions you are suggesting look great. Please go ahead and revise your paper as you mentioned.

Please attend to all of the reviewers' comments and ensure that you clearly highlight all changes made in the revised manuscript. Please avoid using 'Tracked changes' in Word files as these are lost in PDF conversion. I should be grateful if you would also provide a point-by-point response detailing how you have dealt with the points raised by the reviewers in the 'Response to Reviewers' box. If you do not agree with any of their criticisms or suggestions please explain clearly why this is so.

Second decision letter

MS ID#: DEVELOP/2021/199629

MS TITLE: Wnt/beta-catenin signalling is dispensable for adult neural stem cell homeostasis and activation
AUTHORS: Sophie H L Austin, Rut Gabarró-Solanas, Piero Rigo, Oana Paun, Lachlan Harris, Francois Guillemot, and Noelia Urbán
ARTICLE TYPE: Research Article

I am happy to tell you that your manuscript has been accepted for publication in Development, pending our standard ethics checks.

© 2021. Published by The Company of Biologists under the terms of the Creative Commons Attribution License (https://creativecommons.org/licenses/by/4.0/).
